# Factors influencing subjective well-being in individuals with functional dyspepsia — a path analysis of sex and psychological factors

**DOI:** 10.3389/fmed.2026.1728748

**Published:** 2026-01-30

**Authors:** Mile Volarić, Emil Babić, Tomislav Kurevija, František Babič, Nikola Volarić, Ljiljana Trtica Majnarić

**Affiliations:** 1 Clinical Department of Gastroenterology and Hepatology, University Clinical Hospital Mostar, Mostar, Bosnia and Herzegovina; 2 Faculty of Medicine, University of Mostar, Mostar, Bosnia and Herzegovina; 3 Faculty of Medicine, J. J. Strossmayer University of Osijek, Osijek, Croatia; 4 Faculty of Electrical Engineering and Informatics, Technical University of Košice, Košice, Slovakia; 5 Faculty of Dental Medicine and Health, J. J. Strossmayer University of Osijek, Osijek, Croatia

**Keywords:** functional dyspepsia, moderating effect of sex, psychosocial factors, subjective well-being, symptoms

## Abstract

**Background:**

Functional dyspepsia (FD) is a common gastrointestinal (GI) disorder which significantly impacts quality of life and subjective well-being (SWB). Psychosocial factors have been linked to FD symptoms, which places this disorder among the “disorders of gut-brain interaction.” Recent studies suggest notable sex differences in symptom expression and the level of disruption of daily activities. This study aims to explore the impact of sex on associations between psychological factors and SWB in individuals with FD.

**Methods:**

The study included 191 adults referred to their first endoscopic examination of the upper GI tract due to dyspeptic symptoms. Patients completed validated measures assessing GI symptoms, GI and extra-GI comorbidities, health-related habits, psychological traits (somatization, stress resilience), and indicators of SWB (life satisfaction, positive and negative experiences). Multiple regression and hierarchical block regression analyses were conducted to identify predictors of SWB and to examine the potential moderating role of sex and psychological factors. Subsequently, path analysis was conducted to explore potential causal pathways among sex, psychological variables and SWB.

**Results:**

Participants displayed relatively homogeneous characteristics according to sex. Two main subgroups were identified: a larger group of highly educated, working-age individuals and a smaller group of older adults (>60 years) with higher comorbidity levels. Psychological factors, stress resilience and somatization emerged as the strongest predictors of SWB, while health and lifestyle factors had modest effects. Sex was identified as a significant determinant of SWB in the complex hierarchical model, but only after controlling for somatization and stress-resilience. The path model indicated that other sex-related factors may also influence SWB.

**Conclusion:**

Results pointed toward the need to involve psychological constructs like somatization and stress resilience in studies examining SWB in individuals with FD. The importance was highlighted of examining the gender-related (socially conditioned) factors associated with SWB, and the need to separately assess these factors in older and younger individuals with FD (<60, ≥60). The study revealed a complex interactive network between age, gender, and SWB-related factors, supporting the biopsychosocial model of FD. However, identifying a suitable methodological framework to elucidate these complex relationships remains a challenge.

## 1 Introduction

Functional dyspepsia (FD) is a common gastrointestinal (GI) disorder in adult population (1). The recently published meta-analysis, based on Rome IV (the last update of the Rome criteria), mentioned the pooled prevalence of 6.8% (95% CI 5.8–7.9). It was higher in developing than developed countries, and in women compared to men (2). In spite of its high prevalence, this disorder is difficult to accurately diagnose, since specific biomarkers and an optimal diagnostic workup are missing (3). Diagnosis is based on symptom clustering, which is an uncertain approach (4). The only way to rule out organic causes of dyspepsia is by performing upper endoscopy. However, it is not a feasible method, since the prevalence of clinically significant endoscopic findings is low. Based on data from systematic reviews and meta-analyses, FD accounts for 70–80% of individuals with chronic dyspepsia (5, 6).

This disorder can also be registered in children over eight years of age and adolescents, as a part of the spectrum of functional GI disorders (7). Clinical recognition is more difficult than in the adults due to the heterogeneity and overlap of symptoms. Pathophysiology is less understood, since scientific evidence is less accessible.

According to Rome IV, the diagnosis of FD is based on the presence of at least one of four bothersome symptoms: postprandial fullness, early satiation, epigastric pain, and epigastric burning, in the absence of organic disease confirmed through endoscopic evaluation (8). These symptoms are divided into two main FD subtypes: postprandial distress syndrome (PDS), which is linked to meal intake, and epigastric pain syndrome (EPS), which occurs independently of meals. The mixed type shows features of both subtypes (4, 9). Recognizing these symptoms requires an accurate understanding of symptom descriptors by the patient. Another fact that makes diagnosis of FD difficult is that dyspeptic symptoms often coexist with other, less specific GI symptoms, such as bloating in the upper abdomen, nausea, and belching. Heartburn – a typical reflux symptom, also often coexists with dyspeptic symptoms. FD is one of the most common of “Disorders of the Gut–Brain Interaction,” previously known as functional gastrointestinal (GI) disorders (9, 10). FD often overlaps with some of these disorders; especially common is a coexistence of FD with Irritable bowel syndrome (IBS).

**Figure 1:**
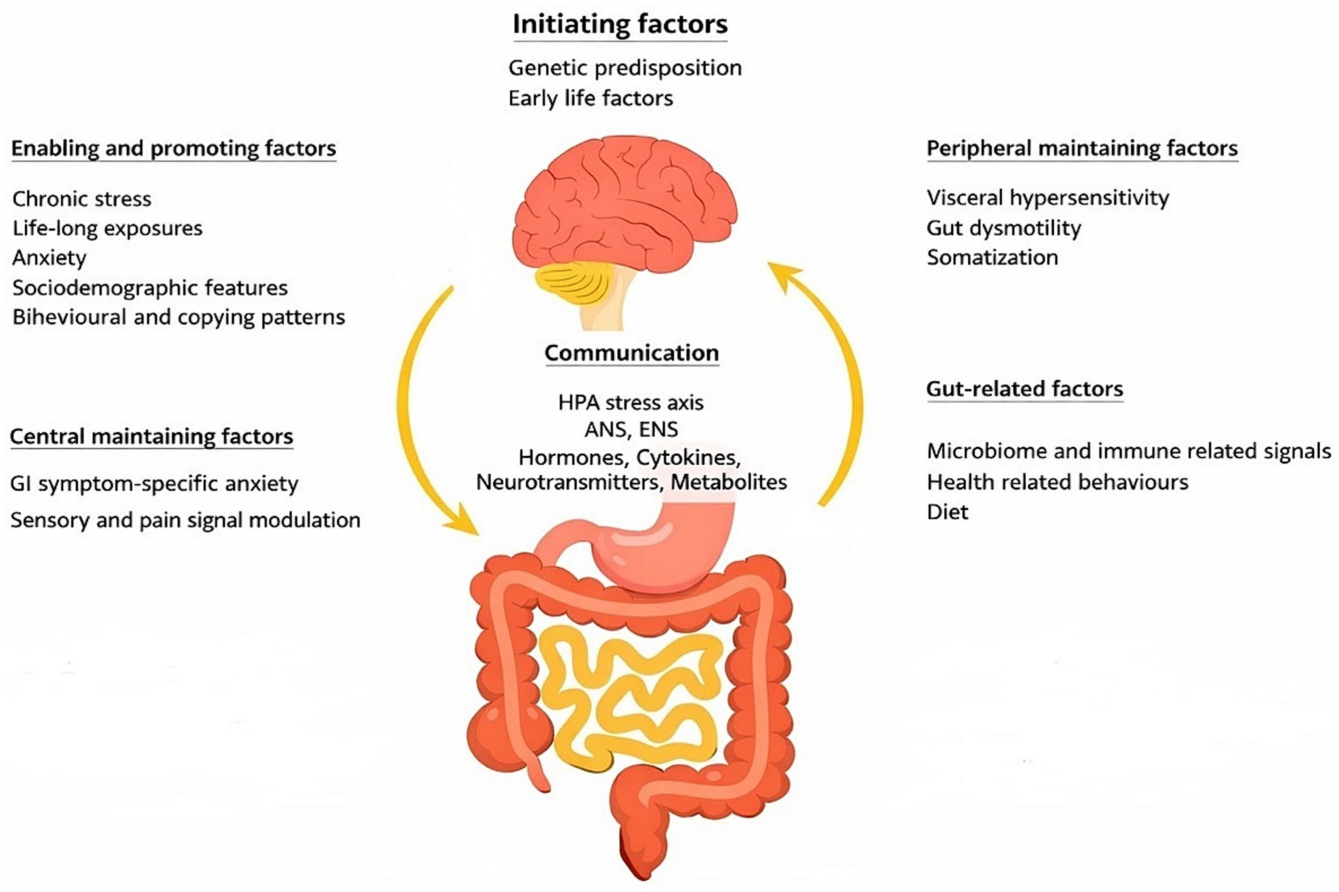
The gut-brain interaction model. The communication channels between the gut and the brain include soluble components, such as hormones, cytokines, neurotransmitters, and metabolites, as well as the wired components, such as the autonomic nervous system (ANS) and the enteric nervous system (ENS). Through this communication network, gut-related signals, in particular of immune and microbiome origins, can communicate with the brain and influence mental health and behavior. And vice versa, altered brain circuits, either directly, through the Hypothalamic–Pituitary–Adrenal (HPA) stress axis, or indirectly, by changes in behavioral patterns, may influence gut motility, visceral sensitivity, mucosal permeability and immune function, and the composition of gut microbiome. Anxiety associated with an exposure to chronic stress, as well as GI symptom-specific anxiety, can amplify somatization and symptom perception.

The concept of the Gut-Brain Interaction in the development of functional GI disorders has evolved from the growing evidence indicating that symptom features in these disorders are determined by the multiple pathophysiological processes. These processes include abnormal gut motility, visceral hypersensitivity, mucosal immune dysregulation, alterations of gut microbiome content and dysregulation of the central nervous system (CNS) signal processing (9). The system view of these disorders led to the model that considers a bidirectional communication between the gut and the brain, based on the mentioned processes, by which these organs regulate the functions of each other (11, 12) (Figure 1). This model aligns with the observation that functional GI disorders are often linked with mental health issues and chronic pain (10).

Due to its high prevalence, uncertainties in diagnosis, and a lack of efficient treatments, FD is considered a major public health concern (3, 5, 10). This disorder also significantly impairs quality of life (QL) and subjective well-being (SWB), even more than it is in dyspepsia caused by organic GI diseases (5). It is considered that identifying risk factors and linking them to pathophysiological mechanisms will allow improvements in the diagnosis and treatment of this disorder, providing a framework for its understanding from a biopsychosocial perspective (13).

Multiple risk factors for chronic dyspepsia have been identified in early population studies, before application of ROME III and IV criteria. These include psychosocial factors such as anxiety, depression, lower socioeconomic status and education, altered stress sensitivity or lack of emotional support, and unhealthy behaviors, such as smoking and excessive alcohol consumption (14, 15). Nonsteroidal anti-inflammatory drugs (NSAIDs) and *Helicobacter pylori* (HP) infection have also been mentioned as contributing factors, but with a moderate effect.

A similar spectrum of factors has been identified in relation to FD, with psychosocial factors being given a dominant role in the onset and modification of FD symptoms (5, 8). Also more recent population studies, based on the application of ROME III and IV criteria, as well as some well-controlled cohort studies of individuals with FD referred to specialist gastroenterologists, in which the diagnosis of FD was confirmed by endoscopic examination, showed that in people with FD there is a higher prevalence of psychological disorders, compared to both the general population and people with organic diseases of the GI system, including disorders such as anxiety, depression, psychological distress, sleep disorders, hostility and alexithymia. The presence of these disorders significantly influences their QL (16–19). In prospective follow-up, lower levels of anxiety and sleep disturbances were shown to predict better clinical outcomes in these individuals (20).

In some studies, irregular mealtimes and frying as a method of food preparation, highly spicy food, as well as some specific food ingredients were mentioned as factors associated with FD (21). However, the possible causal relationship of dietary factors with the onset or maintenance of FD symptoms is poorly scientifically based. In a recent large world-wide study encompassing low- and middle-income countries, modifying factors associated with FD were identified as: higher stress, chronic fatigue, smoking, deviations from normal BMI values (decreased or increased), too few or too many hours of sleep, and previous Covid-19 infection, while older age, female sex and the presence of chronic diseases were identified as non-modifying factors (22).

Most of these studies were observational and often poorly controlled, which makes difficult to draw conclusions from them about a possible causal association of significant factors with FD. In order to make easier to clinicians to manage FD in clinical practice, United European Gastroenterology (UEG) and European Society for Neurogastroenterology and Motility (ESNM) issued a consensus paper in 2020, based on grading the available evidence and voting upon a list of statements by the expert group (23). Of risk factors for FD, for which consensus was achieved, there were only female sex, acute GI infections, and anxiety. The risk factors for which consensus was not achieved, like age at which there is a peak incidence of FD, NSAID use, antibiotic therapy, depression, and smoking, were qualified as areas in need of future research. It is also stated in this report that in individuals with FD and normal macroscopic findings at endoscopy, testing on HP infection, and treating with eradication therapy those with a positive test result is necessary to rule out cases of HP-associated dyspepsia, and to confirm the diagnosis of FD.

This consensus report showed that anxiety is consistently supported by a number of cross-sectional and population-based studies as associated with FD in. In longitudinal studies, anxiety was shown to precede dyspeptic symptoms, thus predisposing to the onset of FD (24). Moreover, in a conveniently planned longitudinal study, Koloski et al. showed that a coexistence of anxiety and FD may start so that dyspeptic symptoms precede anxiety or that anxiety precedes dyspeptic symptoms, thus providing a support to the concept of gut-brain interaction (25). There are also evidence supporting a view that childhood trauma and early life experience can initiate a cycle of interfering between mood disorders and GI symptoms later in adulthood, with the modulating effect of anxiety trait, fitting tightly into the context of the biopsychosocial model of GI disorders (13, 26) (Figure 1).

In this context, individuals with FD were found to exhibit increased stress levels, which correlates with symptom severity (27). They have also been observed to have an above-average tendency for somatization (28). Somatization is manifestation of physical symptoms in those experiencing psychological distress. In individuals with FD, somatization was found to correlate more strongly with dyspeptic symptom severity than objective measures of gastric function (29, 30). Apart of the reaction to stress, GI-specific anxiety, reflecting how much fear and worry about GI symptoms an individual express, can amplify somatization and thus, also, symptom severity (31). Symptom-specific anxiety has been identified as a key mechanism in maintaining disorders of gut-brain interactions, operating through mental mechanisms such as cibophobia (fear of some kind of food), symptom hypervigilance (excessive focusing on symptoms) and symptom catastrophization (perception that symptoms are worse than they are) (32) (Figure 1).

Taken together, the impact of psychosocial factors on dyspepsia-related symptoms may manifest through variations of how environmental exposures affect the GI tract or in one’s perception of symptoms (33). In addition, psychosocial factors can negatively affect SWB, which may lead to unhealthy coping habits (14). A key element in these complex interactions is the alteration in the activity of the Hypothalamic–Pituitary–Adrenal (HPA) stress axis and autonomic nervous system (ANS), which enhances bidirectional communication between the brain and the gut through neurological, endocrine, and immunological pathways (29) (Figure 1).

Recent studies have shown notable differences in how men and women with FD experience symptoms and manage daily activities. Women often face more disruptions and exhibit more somatization and EPS symptoms compared to men (19, 34). There is an increasing interest in research on sex- and gender-related differences in factors associated with FD, and other disorders of gut-brain interaction, in general (6, 35–37). These differences can be biological, such as how sex hormones influence GI organs and brain function, or stem from social and behavioral norms that differ between men and women, which is termed sex-related differences (35, 38). For example, studies indicate that women tend to react more intensely to stressful situations than men, likely due to differences in their neuroendocrine stress response (39, 40). However, sociocultural and gender-based factors also contribute to stress response differences between sexes (41). These variations may result from differences in exposure to stressors and how individuals cognitively appraise and emotionally or behaviorally cope with stress (42–44). Recent research distinguishes between susceptibility to stressors and vulnerability to coping strategies, providing insight into the disparities in health outcomes between men and women (45–47). For instance, women are more likely to use emotion-focused coping styles and experience depressive reactions more often than men (41, 48, 49).

In fact, the response to chronic stress results from a complex interaction of biological and psychosocial factors, especially those related to gender (50). Various sociodemographic factors, such as age, sex, gender, socioeconomic status, education level, and lifestyles, influence how individuals respond to stress and contribute to health disparities based on gender (51). Moreover, research has demonstrated that sociodemographic variables can affect a person’s vulnerability to unhealthy lifestyles and environmental risk factors, helping to explain the pathway from these risks to the development of disease (52).

Although risk factors for chronic dyspepsia and FD have already been defined, the impact of sex on the relationship between certain factors and reduced life satisfaction and functional efficacy, measured by SWB, in individuals with FD has not yet been explored. This study aims to address that gap. A better understanding of these relationships would enhance our comprehension of the heterogeneity of FD and guide therapeutic approaches, which may differ for men and women.

## 2 Methods

### 2.1 Study design

This is a clinical study with a cross-sectional design, conducted and reported in accordance with the STROBE guidelines (53). The research took place in the broader area of Mostar, Bosnia and Herzegovina, and lasted over two years, from 2022 to 2024. In this area, Croatian is an official language.

The study included 191 participants (M: W = 40%:60%) with a median age of 42 (M = 43, W = 41). The obtained sample size was greater than the sample size estimated by the power analysis, ensuring adequate statistical power for the analyses. The distribution of participants according to sex allowed for examination of the effect of sex on associations of selected variables with the outcome measures. Power analyses were conducted in R.

### 2.2 Participants

**Figure 2:**
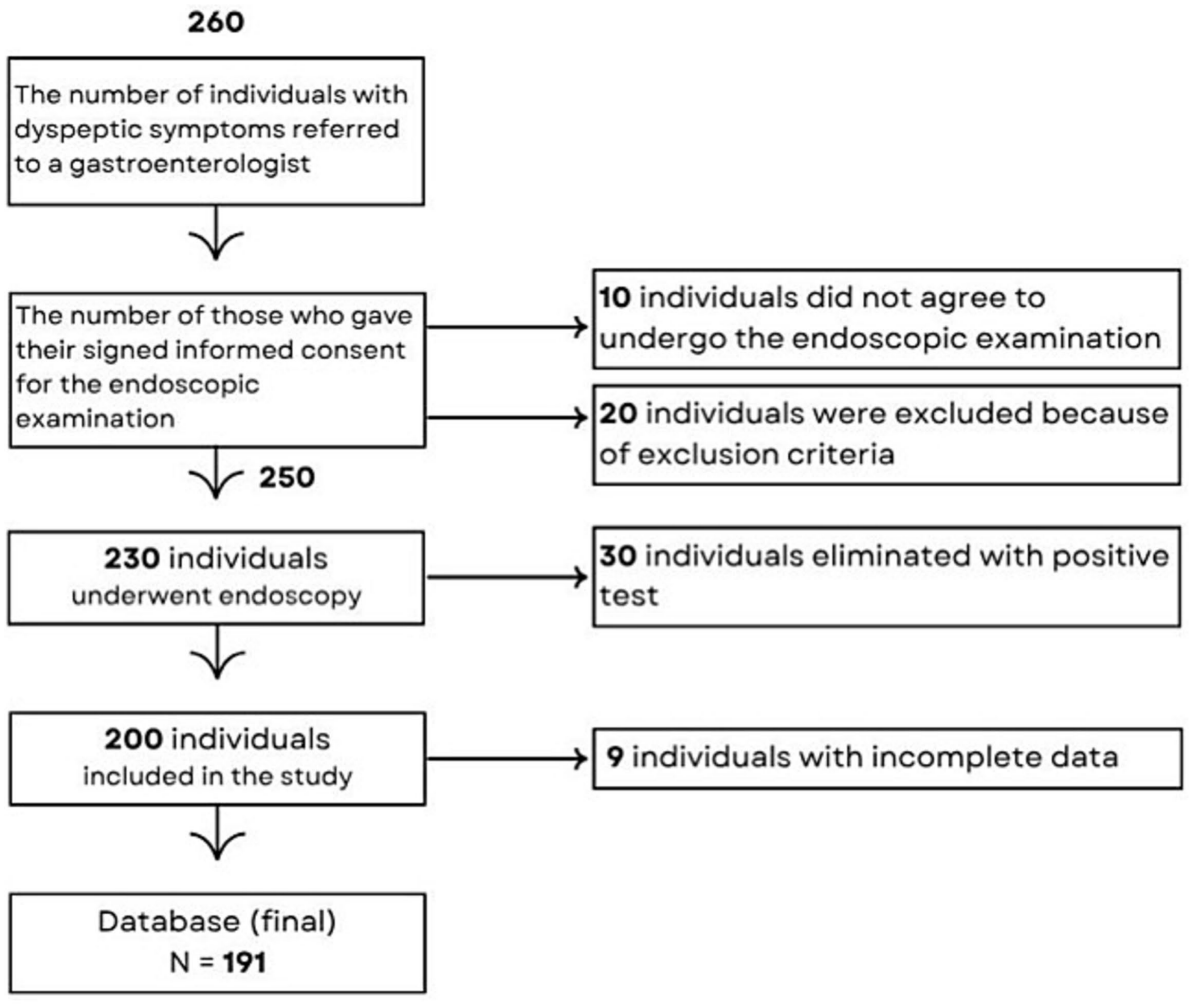
A flowchart diagram of participant selection process.

The study involved adults aged 18 and older referred for their first endoscopic examination of the upper GI tract due to dyspeptic symptoms. They were all informed about the purpose of the study. Those who agreed to participate and signed the informed consent form were interviewed before undergoing an endoscopic examination. Only those with negative endoscopic results were included in the study (Figure 2).

Exclusion criteria were psychological or cognitive disorders that hinder communication, poor overall health, previous GI surgeries, inflammatory or autoimmune intestinal diseases, pancreatic disorders, severe liver conditions, or unrelated malignant diseases. Participants with confirmed cholelithiasis or related symptoms, those diagnosed with IBS, and individuals with heartburn who also showed FD symptoms but had a negative endoscopic exam were included. These selection rules follow the recommendations of the Rome IV criteria (8).

The data on testing of HP infection were obtained from participants. In the area of research (the canton in Bosnia and Herzegovina where Croats live), medical doctors use the Croatian national guidelines for diagnostics and treatments of HP infection (54). The “test-and-treat” strategy is recommended for individuals under 50 years of age with dyspeptic symptoms of a longer duration, and an endoscopic examination for those older than that age and those with new onset of alarm symptoms, irrespective of age. These recommendations rely on the guidelines of the Maastricht IV/Florence Consensus Conference for populations with a prevalence of HP infection over 20%, and recommendations from the last Consensus Conference (Maastricht VI) did not change in this regard (55, 56).

According to the principal researcher’s experience, individuals under 50 years of age with dyspeptic symptoms who were referred by their family doctors for an endoscopic examination already had results of the non-invasive stool antigen test (SAT). For those in whom SAT was positive, a family doctor performed eradication. Individuals who felt better after eradication did not come to the gastroenterologist for an endoscopic examination, only those with persistent symptoms did.

A subsequent analysis of the research data showed that the number of participants who had taken SAT before the endoscopic examination was 116 (60.7% of a total number of participants) (Table 1). Of them, 29 individuals (25%) had confirmed HP infection and received eradication therapy, but did not respond, so that they have been referred to the gastroenterologist. The data on whether post-eradication SAT was performed by family doctors were not available. The number of participants who have not taken SAT before their referral to the gastroenterologist was 75. They were mostly those older than 50.

**Table 1:** FD-related symptoms, other GI symptoms, GI and other comorbidities.

Variable	Men (*N* = 76)	Women (*N* = 115)	Total (*N* = 191)	*p* (Sex differences)
FD Subtype				> 0.05
EPS	8 (10.5%)	19 (16.5%)	27 (14.1%)	
PDS	43 (56.6%)	67 (58.3%)	110 (57.6%)	
Mixed	25 (32.9%)	29 (25.2%)	54 (28.3%)	
FD symptoms presentation				
Symptom intensity (Likert scale 1–5; M ± SD)	2.38 ± 0.89	2.51 ± 1.00	2.46 ± 0.96	> 0.05
Symptom frequency				> 0.05
Every day	14 (18.4%)	24 (20.9%)	38 (19.9%)	
Several times a week, not daily	33 (43.4%)	45 (39.1%)	78 (40.8%)	
Once a week	16 (21.1%)	16 (13.9%)	32 (16.8%)	
2–3 times a month	8 (10.5%)	19 (16.5%)	27 (14.1%)	
Once a month	5 (6.6%)	11 (9.6%)	16 (8.4%)	
Symptom duration				**< 0.05**
3–6 months	17 (22.4%)	46 (40.0%)	63 (33.0%)	
6 months to 1 year	27 (35.5%)	31 (27.0%)	58 (30.4%)	
1–3 years	21 (27.6%)	25 (21.7%)	46 (24.1%)	
3–5 years	5 (6.6%)	4 (3.5%)	9 (4.7%)	
More than 5 years	6 (7.9%)	9 (7.8%)	15 (7.9%)	
Alarm symptoms (most common: unexpected weight loss, family history of upper GI cancer)				> 0.05
Present	27 (35.5%)	32 (27.8%)	59 (30.9%)	
One	18 (23.7%)	21 (18.3%)	39 (20.4%)	
Two and more	9 (11.8%)	11 (9.5%)	20 (10.5%)	
Other GI symptoms (most common: belching, bloating/digestive issues)				> 0.05
Present	66 (86.8%)	98 (85.2%)	164 (85.9%)	
One	12 (15.8%)	13 (11.3%)	25 (13.1%)	
Two and more	54 (71.0%)	85 (73.9%)	139 (72.8%)	
Other GI diseases (most common: IBS, Cholelithiasis)				> 0.05
Present	27 (35.5%)	29 (25.7%)	56 (29.6%)	
IBS	18 (23.7%)	18 (15.9%)	36 (19.0%)	> 0.05
*H. pylori* infection				> 0.05
Total number of participants tested	42 (55.3%)	74 (64.3%)	116 (60.7%)	
Total number of those with positive test results and who received an eradication therapy	10 (13.2%)	19 (16.5%)	29 (15.2%)	
Number of somatic and neurological comorbidities				> 0.05
0	34 (44.7%)	53 (46.1%)	87 (45.5%)	
1	25 (32.9%)	38 (33.0%)	63 (33.0%)	
Two and more than two	17 (22.4%)	24 (20.9%)	41 (21.5%)	
Hypertension	21 (27.6%)	28 (24.3%)	49 (25.7%)	> 0.05
Allergic rhinitis/asthma	19 (25.0%)	17 (14.8%)	36 (18.8%)	> 0.05
Autoimmune thyroid disease	1 (1.3%)	17 (14.8%)	18 (9.4%)	**< 0.01**
Urticaria/eczema tendency	6 (7.9%)	12 (10.4%)	18 (9.4%)	> 0.05
Psychiatric disorders present (most common: anxiety)	24 (31.6%)	38 (33.0%)	62 (32.5%)	> 0.05
Anxiety	19 (25.0%)	32 (27.8%)	51 (26.7%)	> 0.05
Family history of mental illnesses	1 (1.3%)	1 (0.9%)	2 (1.0%)	> 0.05
Psychological problems in early childhood or adolescence present	1 (1.3%)	5 (4.3%)	6 (3.1%)	> 0.05

Biopsies were not taken from respondents during the endoscopic examination, unless in case of the pathologic macroscopic findings (and these individuals were excluded from the study) (Figure 2). This approach was based on cost–benefit considerations. To participants without SAT at the endoscopy, a gastroenterologist recommended to take that test and to show him a result at the follow-up examination, bearing in mind that in older individuals this test may be of diminished sensitivity (57).

A number of participants who had not taken the SAT before endoscopy was 75. Since invasive testing for HP infection has not been planned in participants with negative endoscopic findings, we tried to estimate the potential number of those who might have HP-associated dyspepsia, and who could, accordingly, be wrongly classified as FD. For this purpose, we used the data on the prevalence of HP infection from the large Croatian study performed on dyspeptic patients undergoing routine endoscopy and by using histological and immunohistochemical diagnostic methods (58). The prevalence of HP infection in this study was determined to be 41%. When we applied this percentage on the number of 75 participants, corrected for the expected percentage of those who might be responsive to eradication therapy, which according to the evidence amounts about 10%, we have come to the number of maximally 3 individuals who potentially would not be accurately classified as to have FD (56). We considered this number not relevant for the study results.

### 2.3 Ethical statement

The study complied with the World Medical Association Declaration of Helsinki 2013. It was approved by the Ethics Committee of the *University Clinical Hospital* Mostar (No. 1190/22) and the Faculty of Medicine, the Josip Juraj Strossmayer University of Osijek (No. 641–01/18–01/01).

### 2.4 Data

This manuscript represents a part of a larger investigation into the psychosocial factors associated with FD, with an emphasis on a sex difference. It included a self-reported questionnaire and standardized psychological tests placed online via Google. A gastroenterologist conducted the interviews and recorded the responses.

The questionnaire contained data on dyspeptic and other GI symptoms, GI and extra-GI comorbidities, lifestyles and health-related habits (including medications affecting GI and mental functions), and sociodemographic characteristics (Tables 1, 2). Subgroup differences according to age (<60, ≥60) in some relevant variables are presented in Tables 3, 4.

**Table 2:** Socio-demographic data (*n* = 191).

Variable	Men (*N* = 76)	Women (*N* = 115)	Total (*N* = 191)	*p* (Sex differences)
Sociodemographic data				
Age (M ± SD)	45.6 (12.5)	43.4 (14.6)	44.3 (13.8)	> 0.05
Level of education (“more than 12 years”)	50 (65.8%)	82 (71.3%)	132 (69.1%)	> 0.05
Employment status (“employed”)	54 (71.1%)	76 (66.1%)	130 (68.1%)	> 0.05
Occupation (among the employed)				**< 0.01**
Office-related jobs	36 (62.1%)	55 (56.1%)	91 (58.3%)	
In services and crafts	9 (15.5%)	16 (16.3%)	25 (16.0%)	
Homemaker or domestic work	1 (1.7%)	20 (20.4%)	21 (13.5%)	
Manual work	11 (19.0%)	7 (7.1%)	18 (11.5%)	
Agriculture	1 (1.7%)	0 (0.0%)	1 (0.6%)	
Marital status				> 0.05
In marital union	50 (65.8%)	69 (61.1%)	119 (63.0%)	
Single	16 (21.1%)	32 (28.3%)	48 (25.4%)	
Divorced	5 (6.6%)	4 (3.5%)	9 (4.8%)	
Widowed	5 (6.6%)	8 (7.1%)	13 (6.9%)	
Children YES	55 (72.4%)	71 (61.7%)	126 (66.0%)	> 0.05
Work-related stress conditions present (most common: constant communication with people)	58 (76.3%)	76 (67.3%)	134 (70.9%)	> 0.05
Conflicted family relationships YES	14 (18.4%)	9 (8.0%)	23 (12.2%)	**< 0.05**
Conflicted work/close-contact relationships YES	13 (17.1%)	12 (10.6%)	25 (13.2%)	> 0.05
Other adverse life circumstances present (most common for men: debt; for women: illness or unemployment of household member)	29 (38.2%)	28 (24.8%)	57 (30.2%)	**< 0.05**
Stressful events in past 6–12 months (number of stressful events)				> 0.05
0	43 (56.6%)	58 (50.4%)	101 (52.9%)	
1	25 (32.9%)	50 (43.5%)	75 (39.3%)	
2	8 (10.5%)	7 (6.1%)	15 (7.9%)	
Lifestyles and health-related habits				
Smoking				**< 0.001**
Current smokers	42 (55.3%)	33 (28.7%)	75 (39.3%)	
Former smoker	15 (19.7%)	22 (19.1%)	37 (19.4%)	
Non-smoker	19 (25.0%)	60 (52.2%)	79 (41.4%)	
Alcohol consumption				**< 0.001**
Almost never or rarely	31 (40.8%)	91 (79.1%)	122 (63.9%)	
Often, not daily	33 (43.4%)	22 (19.1%)	55 (28.8%)	
Frequently, several times a week	12 (15.8%)	2 (1.7%)	14 (7.3%)	
Coffee consumption (No)	4 (5.3%)	10 (8.7%)	14 (7.3%)	> 0.05
Tendency to use opioid substances (No)	74 (97.4%)	112 (99.1%)	186 (98.4%)	> 0.05
Night work or study (Yes)	25 (32.9%)	45 (39.8%)	70 (37.0%)	> 0.05
Physical activity: Sedentary lifestyle	27 (35.5%)	21 (18.6%)	48 (25.4%)	**< 0.05**
Unhealthy dietary habits (at least one)	73 (96.1%)	94 (83.2%)	167 (88.4%)	
Number of unhealthy dietary habits (median [Q1, Q3])	3 [2,5]	2 [1,3]	2 [1,3]	**< 0.001**
Meal timing irregularities (at least one)	67 (88.2%)	100 (88.5%)	167 (88.4%)	
Number of meal timing irregularities (median [Q1, Q3])	2 [1,3]	1 [1,2]	2 [1,2]	**< 0.01**
Number of previously applied dietary interventions to reduce or eliminate symptoms (median [Q1, Q3])	2 [1,2]	1 [1,2]	2 [1,2]	> 0.05
Continuous aspirin use or long-term NSAID use (Yes)	20 (26.3%)	26 (23.0%)	46 (24.3%)	> 0.05
Long-term use of other medications with adverse effects on the GI tract (most common: antibiotics)	47 (61.8%)	70 (61.9%)	117 (61.9%)	> 0.05
Total number of medications				> 0.05
0	29 (38.2%)	43 (38.1%)	72 (38.1%)	
1	15 (19.7%)	30 (26.5%)	45 (23.8%)	
2	13 (17.1%)	16 (14.2%)	29 (15.3%)	
Three and more than three	19 (25.0%)	24 (21.2%)	43 (22.8%)	
Anthropometric measures				
BMI (kg/m2) (% with BMI ≥ 25)	79.7%	43.5%	57.7%	**< 0.05**
Waist circumference (% with ≥80 cm for women, ≥ 94 cm for men)	44.6%	52.2%	49.2%	> 0.05

Significant differences between men and women. Tests performed - T Test or Mann–Whitney U for continuous variables, Chi Square for categorical variables.

**Table 3:** Differences in participants’ characteristics according to their age.

Variable	Up to 60 years old (*n* = 163)	60 + years old (*n* = 28)	*p*
Sex/W	97 (59.5%)	18 (64.3%)	> 0.05
Number of alarm symptoms	0.49 ± 1.03	0.50 ± 0.75	> 0.05
Number of other GI symptoms	2.42 ± 1.62	2.71 ± 1.58	> 0.05
Number of diagnoses of GI system	0.37 ± 0.72	0.33 ± 0.48	> 0.05
H.pylori infection	24 (14.7%)	5 (17.9%)	> 0.05
IBS	32 (19.4%)	5 (17.9%)	> 0.05
Anxiety	42 (25.8%)	9 (32.1%)	> 0.05
Number of somatic comorbidities	0.64 ± 0.76	1.68 ± 0.82	**< 0.001**
Hypertension	26 (16.0%)	23 (82.1%)	**< 0.001**
Autoimmune thyroid diseases	17 (10.4%)	1 (3.6%)	> 0.05
Allergic rhinitis or/and asthma	35 (21.5%)	1 (3.6%)	**< 0.05**
Predisposition to urticaria or skin eczema	16 (9.8%)	2 (7.1%)	> 0.05
Somatization	4.96 ± 3.03	7.82 ± 4.00	**< 0.001**
Stress resilience	3.07 ± 0.61	2.78 ± 0.62	**< 0.05**
Satisfaction with Life Scale	23.49 ± 6.30	19.96 ± 5.48	**< 0.01**
Scale of Positive Experience	21.08 ± 3.79	18.96 ± 2.88	**< 0.01**
Scale of Negative Experience	16.67 ± 3.21	17.69 ± 2.68	> 0.05
Subjective well-being (standardized)	0.22 ± 2.44	−1.30 ± 1.89	**< 0.01**
Prosperity	41.80 ± 8.86	38.86 ± 9.39	> 0.05

Tests performed - T Test or Mann–Whitney U for continuous variables – presented as M ± SD; Chi-square for categorical variables – presented as absolute and relative frequencies *N* (%).

**Table 4:** Differences in psychological factors according to the age of participants.

Variable	Up to 60 years old (*n* = 163)		*p*	60 + years old (*n* = 28)		*p*
	M (*n* = 66)	W (*n* = 97)		M (*n* = 10)	W (*n* = 18)	
Somatization	4.71 ± 2.93	5.12 ± 3.10	> 0.05	6.00 ± 2.98	8.83 ± 4.20	> 0.05
Stress resilience	3.27 ± 0.57	2.94 ± 0.61	**< 0.001**	2.92 ± 0.49	2.70 ± 0.69	> 0.05
Satisfaction with Life Scale	22.88 ± 6.82	23.91 ± 5.93	> 0.05	21.00 ± 4.42	19.39 ± 6.03	> 0.05
Scale of Positive Experience	19.92 ± 3.90	21.88 ± 3.51	**< 0.01**	18.56 ± 1.24	19.18 ± 3.47	> 0.05
Scale of Negative Experience	16.45 ± 3.38	16.82 ± 3.09	> 0.05	16.44 ± 2.79	18.35 ± 2.45	> 0.05
Subjective well-being (standardized)	−0.19 ± 2.68	0.50 ± 2.24	> 0.05	−0.89 ± 0.90	−1.51 ± 2.25	> 0.05
Prosperity	40.58 ± 9.43	42.65 ± 8.38	> 0.05	40.30 ± 6.83	38.06 ± 10.60	> 0.05

Tests performed - T Test or Mann–Whitney U for continuous variables.

We measured an intensity of dyspeptic symptoms using a 5-point Likert Scale. Although validated questionnaires for dyspepsia severity, such as the Patient Assessment of Gastrointestinal Disorders-Symptom Severity Index (PAGI-SYM) and the Leuven Postprandial Distress Scale (LPDS), are available, we decided not to use them, for several reasons.

For example, PAGI-SYM is a 20-item questionnaire with six subscales and is rather long for the already complex design of this study (59). In addition, it contains a mix of GI symptoms, including also symptoms of organ diseases, such as regurgitation or heartburn, while in this study, we focused on FD-related symptoms. The selection of symptoms for this questionnaire was made in the period before Rome IV has been established and does not align with the current comprehension of dyspeptic symptoms and the way of clustering into subtypes. Validation of this questionnaire was performed on the population with chronic uninvestigated dyspepsia. At least, it requires a new validation procedure, that is, on the sample consisted of well-selected individuals with FD, in terms of how this entity is understood today. The LPDS, however, is narrowly focused on symptoms related to PDS (60).

In contrast to these questionnaires, a Likert Scale is a basic, widely used rating scale in psychometric measurements, and serves as a measure for grading individual items in every standard questionnaire. Obviously, there is a need to create and validate an integrated questionnaire for assessing symptoms severity in people with FD, including also symptom intensity, frequency, and duration.

To evaluate participants’ SWB, we used the Diener Subjective Well-Being Scales, which measure life satisfaction and fulfilment (61, 62). We employed the Resilience to Stress Test (63, 64) to measure one’s capability to recover after facing a stressful situation. To measure the level of somatization, we used the reconstructed original The Patient Health Questionnaire (PHQ-15), making it simpler, as a 9-item version (65, 66) (see Supplementary material 1). In selecting the tests, we have been guided by the fact that they were translated into Croatian and used in our surroundings.

White light endoscopy was performed, as a standard endoscopic method to detect changes like inflammation, ulcers, or early cancers based on the mucosal color changes, surface abnormalities, and altered light reflection (67). Endoscopy included visualization of the whole upper GI tract, that is, esophagus, cardia, fundus in retroflexion, corpus, antrum, duodenal bulb, and descending duodenum. A certified, well-trained endoscopist conducted all endoscopic examinations, adhering to quality performance measures of the European Society of Gastrointestinal Endoscopy (ESGE) (68).

### 2.5 Description of psychological instruments

*The Resilience to Stress Test* is composed of six items, three positive and three negative, assessed using a five-point scale. The average score is calculated after reversing the scores for the three negative items. Higher scores indicate greater resilience.

*The Diener Subjective Well-Being Scale* consists of several scales: The Life Satisfaction Scale, The Scale of Positive and Negative Experiences, and The Flourishing (Prosperity) Scale.

*The Life Satisfaction Scale* contains five items that measure overall life satisfaction using a seven-point scale, with higher scores indicating greater satisfaction.

*The Scale of Positive and Negative Experiences* includes 12 items, divided into two sets of six, measuring positive and negative feelings over the past four weeks, with scores ranging from 6 to 30 for both categories.

*The Flourishing (Prosperity) Scale* has eight items assessing key aspects of functioning, such as positive relationships and a sense of competence, using a seven-point scale. Scores range from 8 to 56, with higher scores reflecting greater success in important life areas.

*The Subjective Well-Being composite* was obtained by centering the results of three scales: Life Satisfaction, Positive Experiences, and Negative Experiences.

*The original PHQ-15* contains 15 somatic symptoms, one of which applies only to women. The respondent needs to rate how much these symptoms bothered them in the past month using a three-point scale, scored as 0, 1, or 2. The total score ranges from 0 to 30, with scores of 5, 10, and 15 indicating mild, moderate, and severe levels of somatization, respectively.

We performed a modified 9-item test, with total scores ranging from 0 to 18, and classified as mild (0–4), moderate (5–8), or severe (9–18). We validated this new PHQ scale by assessing internal consistency and criterion validity through correlations with other study measures, including Diener’s Subjective Well-being and the Resilience to Stress Test (see Supplementary material 1).

### 2.6 Statistical methods

Data were screened for extreme or implausible values, and missing responses within multi-item scales were checked to ensure valid total scores. Absolute and relative frequencies represented categorical data. Differences in categorical variables were analyzed using the Chi-square test or Fisher’s exact test. Continuous data were summarized using arithmetic means with standard deviations and medians with interquartile ranges. Group differences in continuous variables were tested with the independent-samples t-test or Mann–Whitney U test. Differences among multiple groups were assessed using ANOVA or the Kruskal-Wallis test, followed by appropriate *post hoc* procedures.

The reliability of a particular psychological tests was assessed by calculating the internal reliability coefficient, Cronbach’s Alpha (Table 5). The relative frequencies of responses on the Somatization Symptom Questionnaire by the sex of participants are presented in Supplementary material 2.

**Table 5:** The results of psychological testing.

Psychological instrument	Mean	SD	Median	IQR	*p* (Sex differences)	Cronbach alpha α
Somatization	5.38	3.34	5	5	> 0.05	0.77
Men	4.88	2.95	5	3.25		
Women	5.70	3.55	5	5		
Stress resilience	3.03	0.62	3	0.83	**< 0.001**	0.75
Men	3.22	0.57	3.17	0.83		
Women	2.90	0.62	3	0.75		
Satisfaction with Life Scale	22.97	6.30	24	9	> 0.05	0.92
Men	22.62	6.56	23	8.75		
Women	23.19	6.15	24	8		
Scale of Positive Experience	20.77	3.74	20	6	**< 0.001**	0.92
Men	19.75	3.70	18	6		
Women	21.46	3.63	22	6		
Scale of negative experience	16.81	3.15	18	3	> 0.05	0.83
Men	16.45	3.29	17	4		
Women	17.06	3.04	18	3		
Subjective well-being (standardized)	−0.002	2.425	0.173	3.400	> 0.05	
Men	−0.281	2.534	−0.796	3.508		
Women	0.182	2.344	0.540	3.299		
Prosperity	41.37	8.97	43	12	> 0.05	0.96
Men	40.54	9.09	41	12.25		
Women	41.92	8.89	44	11		

Differences between men and women.

This study examined whether sex moderates the relationship between FD-related variables and SWB. Multiple regression analyses incorporated an interaction term for sex. The variables assessed were organized into five categories: socio-demographic characteristics, lifestyle and health-related habits, GI symptoms, GI and extra-GI comorbidities, and psychological factors. Due to limitations identified in the power analysis, not all variables could be included simultaneously in the final analysis. To address this constraint and retain theoretically relevant constructs, stepwise regression was conducted within each conceptual category (Supplementary material 3). Predictors that remained significant within each category were then included in the final model using hierarchical block regression analysis (Table 6).

**Table 6:** The hierarchical (block-wise) regression model of subjective well-being.

Predictors	Block 1 of variables				Block 1 + 2 of variables				Block 1 + 2 + 3 of variables			
	Estimate	SE	t value	*p*	Estimate	SE	t value	*p*	Estimate	SE	t value	*p*
(Intercept)	−1.49	0.93	−1.61	0.108	−0.33	1.25	−0.27	0.791	−4.91	1.46	−3.36	**0.001**
Age	−0.03	0.01	−2.43	**0.016**	−0.02	0.01	−1.34	0.181	−0.02	0.01	−1.22	0.223
Sex (ref. M) – W	−0.09	0.35	−0.26	0.794	−0.08	0.35	−0.23	0.821	0.67	0.32	2.07	**0.040**
Conflicted family relationships (ref Yes) - No	0.97	0.50	1.94	0.054	0.52	0.51	1.02	0.307	0.36	0.44	0.83	0.410
Number of stressful events	−0.46	0.26	−1.78	0.077	−0.30	0.26	−1.16	0.246	0.08	0.23	0.35	0.724
Continuous aspirin use or long-term NSAID use - No	1.45	0.40	3.62	**<0.001**	1.19	0.40	2.98	**0.003**	0.85	0.35	2.39	**0.018**
Sedentary lifestyle (ref. Yes) – No	1.01	0.38	2.65	**0.009**	0.76	0.38	2.01	**0.046**	0.60	0.33	1.83	0.068
Smoker (ref. Yes) – No	0.75	0.35	2.17	**0.032**	0.61	0.35	1.74	0.084	0.30	0.31	0.99	0.325
Symptom frequency					−0.36	0.13	−2.68	**0.008**	−0.19	0.12	−1.59	0.114
Symptom duration					−0.28	0.14	−1.96	0.052	−0.12	0.12	−0.99	0.323
Anxiety (ref. Yes) – No					0.99	0.39	2.55	**0.012**	0.37	0.35	1.04	0.301
Hypertension (ref. Yes) - No					0.22	0.45	0.49	0.624	−0.17	0.39	−0.44	0.658
IBS (ref. Da) - Ne					0.16	0.40	0.39	0.694	0.35	0.35	1.02	0.307
Stress resilience									1.58	0.25	6.23	**<0.001**
Somatization									−0.14	0.05	−2.69	**0.008**
Sample	177				177				177			
R^2^/R^2^ adj.	0.283/0.253				0.356/0.309				0.521/0.479			

SE: Standard Error of a regression coefficient. R^2^/R^2^ - significant changes in explained variance (R^2^) across blocks.

**Figure 3:**
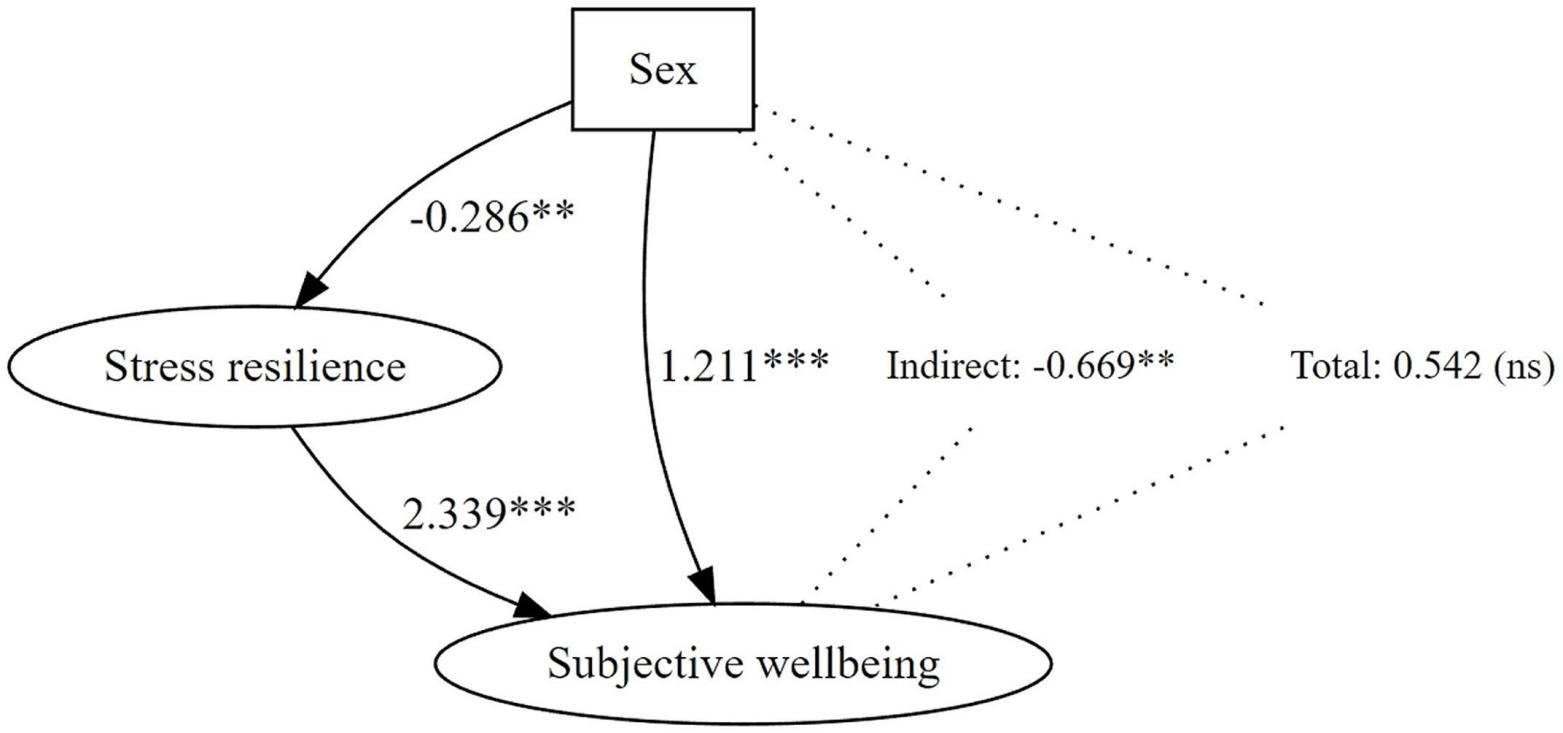
Graph. Trace analysis showing the direct and indirect traces between sexes, subjective well-being, and stress resilience. Direct effect = 1.211*, indirect effect = −0.669**, total effect = 0.542 (insignificant). A significant indirect effect suppresses the direct effect, making the total effect insignificant - a classic example of a suppression effect.

The study utilized path analysis as an extension of multiple regression techniques (Supplementary material 4). This method evaluated hypothetical causal relationships between sex and variables that were significantly associated with SWB in the final block regression model (69). The analysis quantified both direct and indirect effects and illustrated the relationships in a path diagram (Figure 3).

**Figure 4:**
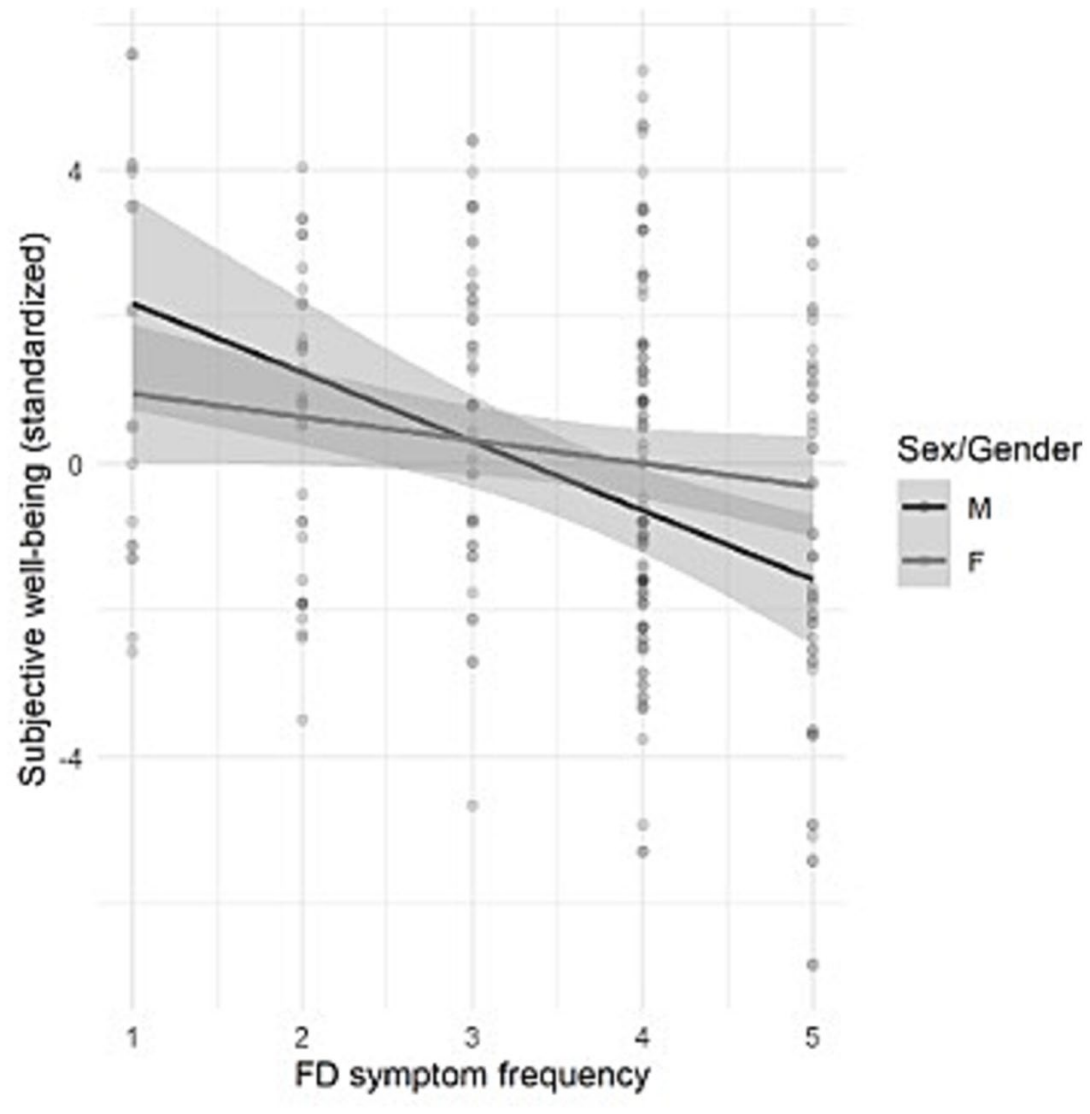
Interaction graph showing the moderating effect of sex on the relationship between FD symptom frequency and subjective well-being.

**Figure 5:**
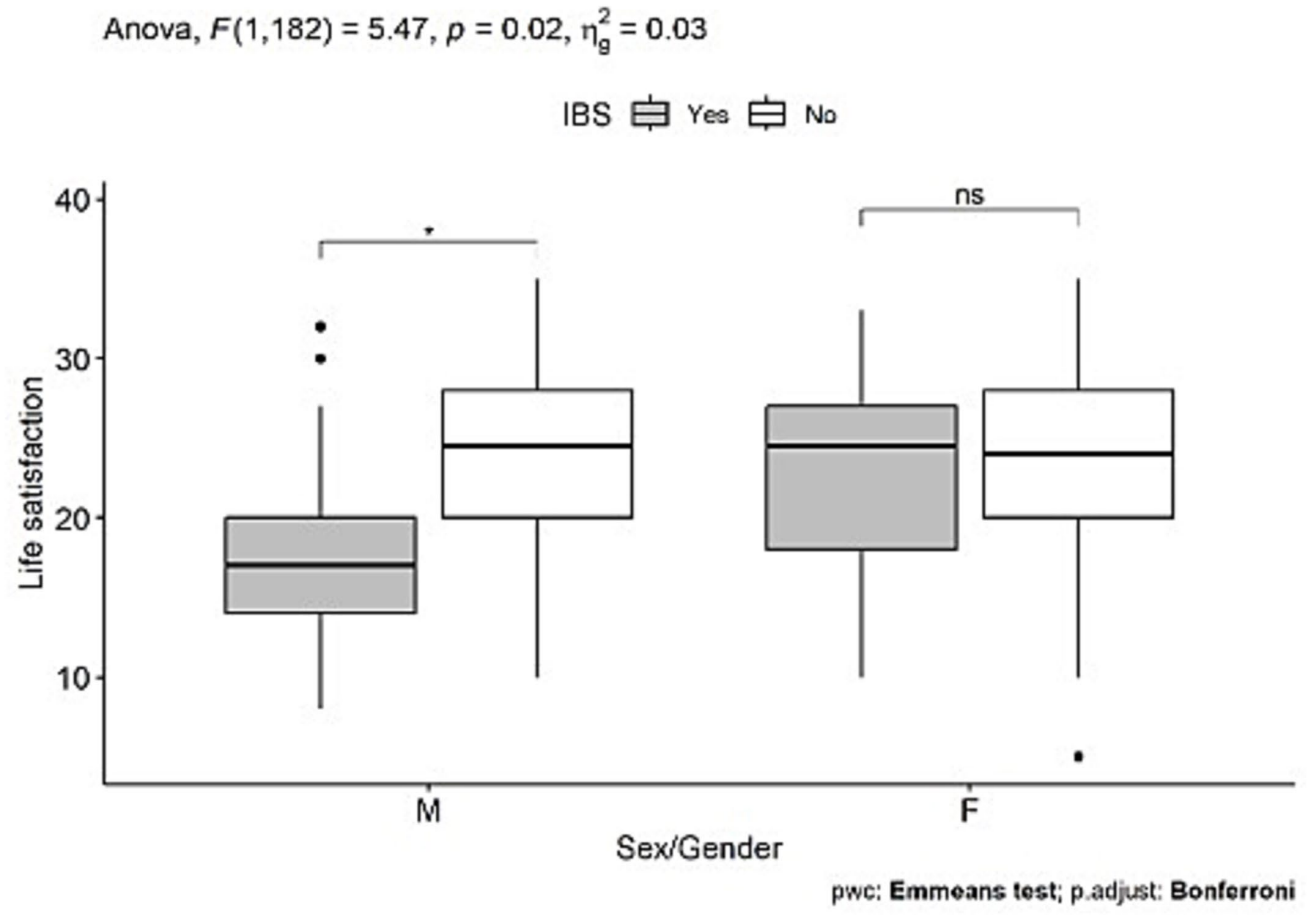
Box-plot graph for the interaction of additional diagnosis of IBS and sex on life satisfaction in individuals with FD.

Additionally, we checked an interaction (moderation) effect of sex on any of the significant variables (interactions included in the models) (Supplementary material 5) (Figures 4, 5).

## 3 Results

Participants are mainly of working age (18 to 60 years), highly educated, and employed in office-related jobs. The second-ranked occupation is in services and crafts. (see Table 2). Their top marital status is married, followed by single. There are no sex preferences in these features.

About 70% of respondents reported experiencing occupational stressors, with frequent communication with third parties being a leading problem. About half of them have experienced at least one stressful situation in the past year. Women most often cited the death or illness of loved ones as their primary source of stress, while men more frequently reported family conflicts as the main stressor.

Unhealthy lifestyle behaviors are highly prevalent, with smoking being the most common. The percentage of current smokers is significantly higher among men than among women. Over one-third of participants report frequent alcohol consumption, a behavior more prevalent in men. All participants also report high coffee intake. Opioid use was reported to be low. More than one-third of the participants work night shifts or study at night. Nearly two-thirds engage in only moderate physical activity or lead mostly sedentary lives, with men being disproportionately affected.

The majority of participants exhibit unhealthy eating patterns, characterized by poor dietary choices and irregular meal timing, with men demonstrating a higher prevalence of these behaviors compared to women. Anthropometric data show that individuals with FD present with normal weight, overweight, or obesity. All participants reported attempting dietary interventions to alleviate FD symptoms before consulting a gastroenterologist.

More than half of the participants reported using medications, primarily for sleep disorders or mental health conditions. Approximately one quarter indicated ongoing therapy with aspirin or long-term use of NSAIDs. No significant sex differences in medication usage were observed.

The most prevalent form of FD was PDS, followed by the mixed type, and then EPS (Table 1). Symptom duration was generally longer in men than in women. Most participants rated their symptom severity as moderate, corresponding to scores of 2 or 3 on the Likert scale. Symptoms occurred frequently, with most individuals experiencing them daily or several times per week.

Approximately one-third of participants reported experiencing alarm symptoms. The most frequently cited concerns included unexpected weight loss and a family history of gastric cancer, with similar prevalence among men and women. Additionally, participants reported a range of other GI symptoms, most commonly two to three distinct symptoms per individual.

One-third of participants received diagnoses of both FD and IBS. Cholelithiasis represented the second most common GI diagnosis, independent of surgical history. Sixty percent of respondents underwent *H. pylori* testing, with 25 % of those receiving positive results.

Over 35% of respondents reported having some mental disorder or a psychological problem. Anxiety was the most common. Over half of the participants had some extra-GI somatic conditions, most prevalently hypertension and immunology-mediated diseases, such as allergic and autoimmune diseases. Over 20% of participants reported having two or more diagnoses, indicating multimorbidity.

As seen in Table 3, participants aged 60 years and older (*n* = 28) did not differ significantly from younger participants (*n* = 163) in sex distribution, gastrointestinal symptoms, or most comorbidities (all *p* > 0.05). However, older participants had a significantly higher number of somatic comorbidities (*p* < 0.001), with hypertension being markedly more common in this group (82.1% vs. 16.0%, *p* < 0.001). Conversely, allergic rhinitis or asthma were more frequent among younger participants (21.5% vs. 3.6%, *p* < 0.05). Older participants also exhibited higher somatization (*p* < 0.001) and lower stress resilience, life satisfaction, positive experiences, and SWB (all *p* < 0.05) (Table 4).

Within participants up to 60 years old, women had lower stress resilience (*p* < 0.001) and higher positive experience scores (*p* < 0.01) than men, while no sex differences were observed for other measures. In participants aged 60 and older, no significant sex differences were found for any psychological or SWB variables (all *p* > 0.05).

Both men and women show moderate levels of somatization, without a preference according to gender (see Table 5). The most common complaints were for chronic pain and fatigue (See Supplementary material 2). In contrast to that, men achieved higher scores than women on the Stress Resilience Scale (*p* = 0.0006) (Table 5).

Analysis of the Life Satisfaction Scale reveals no statistically significant difference in overall scores between sexes. Both men and women report moderate life satisfaction, with scores ranging from 21 to 25.

Scores on the Positive Experiences Scale indicate moderate positive affect for both men and women (score range 19–24). However, women report significantly higher frequencies of positive feelings compared to men (*p* < 0.001).

Scores on the Negative Experiences Scale indicates no significant sex differences in the frequency of reported negative experiences. Both men and women demonstrate moderate negative experience scores, ranging from 13 to 18.

Analysis of the Prosperity Scale indicates no statistically significant difference between men and women. All participants are positioned at the threshold between moderate (25–40) and high (41–56) prosperity.

The SWB composite score was calculated by standardizing the results from the Life Satisfaction, Positive Experiences, and Negative Experiences scales. No significant difference was observed between men and women (T-test: t (179) = −1.261, *p* = 0.209).

The number of variables that entered the hierarchical (block-wise) regression model was reduced by a pre-selection procedure including separate models for each group of variables (see Supplementary material 3).

The final model of SWB was obtained by gradually entering variables significant from separate models for each conceptual block in a hierarchical (block-wise) regression analysis (Table 6). The control variables, age and sex, were also entered into the model regardless of their significance. There were three blocks and 15 input variables in total.

First block: Socio-demographic variables + health habits.

Seven predictors: Age + Sex + Family relationships + Number of stressful events + Continuous aspirin use or long-term use of NSAIDs + Sedentary lifestyle + Smoking.

Second block: Symptoms and comorbidities.

Five predictors: Symptom frequency + Symptom duration + Anxiety + Hypertension + IBS.

Third block: Psychological characteristics of the participants.

Two predictors: Stress resilience + Somatization tendency.

In Block 1 (socio-demographic variables, lifestyle, and health-related habits; *F* (7,169) = 9.537; *p* < 0.001), older age was associated with lower SWB (*p* = 0.016), and participants who did not use aspirin/NSAIDs continuously, were physically active, or non-smokers reported higher SWB (all *p* < 0.05). Family conflicts approached significance, while the number of stressful events was not significant. This block explained 25% of the variance in SWB (adjusted R^2^ = 0.253).

Adding Block 2 (symptoms and comorbidities; *F* (12,164) = 7.549; *p* < 0.001) slightly reduced the significance of some lifestyle predictors, while higher symptom frequency was associated with lower SWB (*p* = 0.008), and absence of anxiety predicted higher SWB (*p* = 0.012). Inclusion of health-related predictors in the second block significantly increased explained variance (ΔR^2^ = 0.073, *F* (5, 164) = 3.699, *p* = 0.003), resulting in a model that accounted for 31% of the variance (adjusted R^2^ = 0.309).

The variable “Age” had initially been found as significantly associated with SWB, but after symptoms and comorbidities were included in the model, the effect of age diminished.

Correlation analysis indicated a strong association between age and hypertension (correlation coefficient = 0.6). However, mediation analysis demonstrated that hypertension alone does not mediate this relationship, and no significant interaction effects were identified. These findings indicate that the relationship between age and SWB is attributable to the combined influence of multiple health factors rather than age in isolation (authors’ note).

Finally, Block 3 (psychological variables; *F* (14,162) = 12.57, *p* < 0.001) showed that higher stress resilience strongly predicted greater SWB (*p* < 0.001), and higher somatization predicted lower SWB (*p* = 0.008). After including psychological variables, sex (female) became a significant positive predictor (*p* = 0.040), while other variables lost significance. Adding psychological predictors in the third block significantly increased the explained variance (ΔR^2^ = 0.165, *F* (2, 162) = 27.868, *p* < 0.001), with the final model accounting for 48% of the variance in SWB (adjusted R^2^ = 0.479).

In the full model, psychological factors emerged as the strongest determinants of SWB, explaining variance previously attributed to socio-demographic and health indicators. This suggests that stress resilience and somatization may mediate or “shadow” the effects of socio-demographic and health variables.

Sex was not significantly associated with SWB in the first two blocks. However, after adding psychological factors, sex emerged as a significant variable, indicating that the effect of sex on SWB was suppressed by shared variance with psychological variables. This suggests that sex may have an independent effect on SWB after controlling for psychological factors.

Correlation analyses showed that sex is negatively associated with stress resilience (*r* = 0.25), which itself is positively associated with SWB. Therefore, controlling for stress resilience revealed a unique effect of sex on SWB, previously masked by shared variance.

The path model shows that sex has a significant direct effect on SWB when controlling for psychological resilience to stress. However, the indirect effect through resilience is negative and significant, suppressing the overall effect (Figure 3) (Supplementary material 4). This is a classic example of a suppression effect: psychological resilience masks part of the effect of sex on SWB, which only becomes apparent when resilience is included in the model.

### 3.1 The moderating effect of sex on the association between symptom frequency and subjective well-being

Of the significant variables in the previous models, sex proved to be a significant moderator in the association between the frequency of FD symptoms and SWB (see Supplementary material 5). We treated the frequency of symptoms as a continuous variable on a Likert scale from 1 to 5 (authors` note).

ANOVA indicated that the frequency of FD symptoms has a negative relationship with reported SWB. Increased symptom frequency is strongly associated with reduced SWB in men. For men, the more frequent the symptoms, the lower the SWB (see Graph, Figure 4).

### 3.2 The moderating effect of sex on the association between IBS diagnosis and subjective well-being

The GI and extra-GI comorbidities were also examined on the moderating effect of sex on their associations with SWB (see Supplementary material 5). There was no interaction (moderation) effect.

### 3.3 Examining the effect of IBS diagnosis and sex on subjective life satisfaction (the main component of the subjective well-being construct) using two-way ANOVA

The additional diagnosis of IBS is significantly related to expressed life satisfaction, only in men, while in women, IBS diagnosis does not influence life satisfaction (see Figure 5).

## 4 Discussion

Participants with FD in this study were referred for specialist examination due to worsening symptoms and impaired SWB. They all attempted some dietary interventions to alleviate their symptoms before consulting a gastroenterologist. This selection criterion likely contributed to the relatively homogeneous characteristics among participants, regardless of sex differences. Common features included frequent additional somatic symptoms, like chronic pain and fatigue, other GI symptoms, and comorbidities, with anxiety, hypertension, and IBS being most prevalent. Likely, previous research indicated that impaired QL, which is related to but distinct from SWB, is more frequently observed in individuals with FD in referral settings (70, 71). Factors known from before to influence QL in these contexts are similar to those identified in this study and include overlapping functional GI disorders, particularly the coexistence of FD and IBS, as well as the presence of extra-GI symptoms (70, 71). Psychological factors, anxiety and depression, for which epidemiological studies indicate they are associated with increased symptom severity in individuals with FD, are also more prevalent in referral settings (17, 71).

The participants are primarily of working age, highly educated, and employed individuals. Women dominate over men. A minor group of older individuals (>60 years), characterized by a higher level of comorbidity, can also be noticed. This demographic profile may differ from that in the general population. In this term, epidemiologic studies have identified older age, female sex, and lower education level as risk factors for FD or uninvestigated dyspepsia, but with varying significance depending on study population and geographic regions (5, 14, 37).

There were no significant differences between men and women in average scores achieved on the SWB Scale and the Somatization Scale. Somatization and SWB were shown to be more impaired in older individuals, but they are less represented in the sample. It may be that they are less ready to take an endoscopic examination, more frequently have contraindications for such examination, or are better suited to unpleasant symptoms and, therefore, less likely to seek help. Regarding the latter option, it is known that in older individuals, affective reactions to stressful situations are less intense than in younger individuals (72). Namely, with increasing life experience, most people learn how to rationally cope with stress (73). On the contrary, psychological distress is more likely to manifest with somatic symptoms, cognitive changes, and loss of interest (74). Ultimately, having a chronic disease or a functional disability is stressful and requires efforts to adapt to discomfort, limitations, and emotional distress that come with these conditions.

The difference among respondents was found only in stress resilience, with men showing significantly higher resilience than women. It appears to be a characteristic of younger individuals with FD and is associated with working conditions. Sex was identified as a significant determinant of SWB in the complex hierarchical model, but only after controlling for somatization and stress-resilience. More precisely, a positive correlation with SWB was observed for the female sex. This is likely different from the evidence indicating that women with FD tend to have higher impairment of daily functioning than men with FD (37, 75). These differences may be due to the specific characteristics of the referral population employed in this study.

Further analysis has revealed that this unexpected sex-related association is due to the dual correlation; sex correlates with stress-resilience, which in turn correlates with SWB. Therefore, controlling for stress resilience has revealed a unique effect of sex on SWB, previously masked by shared variance. The path model, as illustrated in Figure 3, further clarifies these relationships. It demonstrates that a significant positive direct effect of sex on SWB is offset by a negative indirect effect. The persistence of a direct effect suggests that additional sex-related factors, apart from stress-resilience, may influence SWB.

In fact, the modeling procedure suggests the presence of a complex interactive network involving age, sex, and several factors associated with FD. These factors belong to different domains, such as socio-demographic and lifestyle characteristics, symptoms, comorbidities, medications, and psychological constructs such as resilience to stress and somatization. This way, this study supports the biopsychosocial model of FD. In addition, the specific characteristics of the study sample and the environmental and cultural context of the study region may contribute to observed sex-related effects on SWB.

Analyzing the hierarchical model retrogradely may clarify the relationships between potential background variables and SWB. The psychological constructs of somatization and stress resilience showed the strongest links to SWB. Adding these psychological variables to the model reduced the explanatory strength of socio-demographic and health-related factors, as psychological constructs explained variance that was previously attributed to those factors. Only the variable “chronic aspirin use or long-term NSAID use” remained significant throughout all model stages, indicating an independent effect on SWB. It may be due to the known harmful effects of these medications on gastric function (76). The pathogenesis of dyspepsia with NSAIDs is not completely understood. It is considered to include both visible and microscopic mucosal injuries and also changes in gastric mechanosensory function (77, 78). Alternatively, this variable may indicate an independent association of age-related accumulation of comorbidities with SWB decline. It is most likely, based on the results of this study, that this variable reflects the increased sensitivity to pain of individuals with FD, in particular those with an overlap of FD and IBS, due to the common presence in participants of somatic symptoms, such as chronic pain and fatigue (79).

Few studies employed a comprehensive design comparable to the present research, limiting opportunities for direct comparison. One study, using a structural equation model, identified somatic symptoms and trait affectivity, defined as a personality feature characterized by emotional responses to stress, as the main factors influencing both physical and mental QL in individuals with FD (80). Another study, with a similar design to this study, and with hospital-based participant selection, found somatization as the principal factor that influenced the physical dimension of QL, and also achieved its effect by mediating a role of abuse history and depression. In contrast, psychosocial variables, such as trait and state anxiety, depression, and both positive and negative affect, were shown to be associated with the mental dimension of QL (81). SWB, used as an outcome in the present study, reflects the emotional and cognitive perception of life and corresponds to the mental aspect of QL.

The diagnoses of anxiety, hypertension, and IBS were the top-ranked participants` comorbidities and were identified as significantly associated with SWB during the pre-selection phase of the model development. Ultimately, only anxiety was found to be significant in the hierarchical regression model, but no longer than somatization and stress-resilience were incorporated into the model. The available evidence may provide a better understanding, linking anxiety and somatization with symptom severity and QL in individuals with FD, with somatization having a mediating role (29, 81). We provided a visual presentation of these relationships in Figure 1 and put them in the context of gut-brain interaction.

The two other previously significant variables in the hierarchical model that lost significance after the model’s adjustment for somatization and stress-resilience are “symptom frequency” and “physical activity.” They can also help clarify the background of the interaction of gender with SWB. Especially, symptom frequency is likely to be important in this context, as sex was shown to moderate its association with SWB in the additional interaction analysis. In this analysis, symptom frequency was negatively associated with SWB in men, but not in women, suggesting a sex-specific vulnerability. That means that symptom burden is likely to have a stronger impact on the subjective mental state in men than in women with FD.

Lower women’s vulnerability to symptom frequency may be due to differences in their coping mechanisms or attitudes toward physical discomfort. Such an assumption is supported by a result in this study showing a significantly higher score on the Positive Experience Scale (a component of SWB) in women compared to men. Thus, by valuing positive experiences better than men do, women are likely to exhibit more optimistic attitudes toward their physical symptoms and life, in general, which could be their protective mechanism against stress. The role of female sex on higher positive affect may be attenuated in older age, when the presence of chronic diseases and a higher level of somatization may make both men and women equally vulnerable to decline in SWB.

Sex-related differences in vulnerability to symptom frequency may account for the observed distribution of this variable between the sexes in the present study. Specifically, there is a relatively higher proportion of men with impaired SWB in this referral group of individuals with FD compared to the general population, where QL impairment is more commonly reported among women (37, 75).

Symptom frequency is closely associated with symptom duration, and both variables were included in the hierarchical model; however, only symptom frequency reached statistical significance, as being more importantly related to SWB. Oppositely, men exhibited a longer symptom duration than women, while no difference in symptom frequency was observed between the sexes. These findings may have a double explanation.

The first option is that men with FD achieve an equal symptom frequency as women with FD after a longer period of symptom duration. This scenario, based on the available evidence, may be as follows. An emotional discomfort related to psychological stress may trigger in men a tendency toward unhealthy coping habits (14, 82). This behavioral pattern is usually more exploited by men than women, as seen also in this study (43). It is particularly comfortable among men in surroundings like those where the study took place, where lifestyles, like smoking and alcohol consumption, are conventionally accepted and considered a typical masculine behavior (83). Because a longer time is needed for the lifestyle and nutritional factors to alter gastric function than it is in women, who typically respond to stress with emotional reactions and anxiety, men are likely to wait longer before achieving symptom frequency to become recurrent or chronic (43).

Alternatively, men with the same symptom frequency and SWB decline as women may wait longer before deciding to report their physical discomfort and ask for help, which points to a sex-related difference in health-seeking behavior.

According to the UEG/ESNM Consensus Report, that is based on evidence systematization and expert panel consensus, dyspeptic subjects who seek healthcare attention have more severe, frequent, and persistent dyspepsia symptoms. Sex-related differences in the relationships between symptom severity and patterns and healthcare seeking behavior, have not been examined so far.

In the context of a poor lifestyle, only smoking and physical activity were included in the hierarchical regression model. However, physical activity was more consistently associated with SBW. This may be because exercise has many health benefits, such as improving mood (84). Poor dietary habits were not associated with FD-related SWB. Currently, the link between dietary patterns and FD remains unclear (85). Still, poor diet and low physical activity can indirectly show up as increased body weight and waist circumference in FD, indicating abdominal obesity linked with metabolic syndrome and hypertension (86). This may be especially the situation with the older part of respondents, who are commonly diagnosed with hypertension.

One more variable, besides symptom frequency, showed an interaction effect of sex on its relationship with SWB. This is the diagnosis of IBS. IBS was negatively linked to life satisfaction (the main component of SWB) in men, but not in women. This result aligns with evidence suggesting that the coexistence of FD and IBS is a key factor affecting QL in referral settings (70, 71). An explanation may be based on evidence that men with both FD and IBS are particularly burdened by psychosocial factors or are more vulnerable to their negative effects (87). This finding could explain the relatively balanced sex-related distribution of IBS diagnoses in this sample, which contrasts to the general population, where IBS is reported to be more common in women (88).

The results of this study highlight the importance of specifying both the source and intensity of stress exposure to assess participants’ responses to stress accurately. Job-related stress was identified as the primary source of stress for participants in this study. However, likely, the combined effect of multiple stressful situations from both work and family domains affects symptom severity and SWB. Several results support this idea. A great part of the participants reported experiencing at least one stressful event in the past year. Variables “Family relationships” and “Number of stressful events” were selected as input variables in the hierarchical regression model. A behavioral pattern that arises in response to stressful situations, such as poor sleep, as also demonstrated in this study, can perpetuate the physiological disturbances initiated by stress (89).

Furthermore, individuals with FD, especially those in referral settings, may exhibit personality profiles that increase their vulnerability to stressors (90). In this sample, the majority of participants demonstrated high levels of ambition, as reflected by their elevated scores on the Prosperity Scale.

This study also underscores the significance of identifying factors that influence sex-related differences in stress responses. Resilience to stress emerged as the primary factor distinguishing men and women in terms of susceptibility to stress. This effect is likely to disappear in older age, when higher somatization and the effect of chronic conditions and polypharmacy may take a dominant role in association with SWB.

Exposure to chronic stress is known to accelerate the development of chronic diseases and disorders such as anxiety, poor sleep, altered metabolic and immune functions, and increased inflammation (91). We discussed this issue in our previous work (86, 92). These mechanisms are especially reinforced among people living in regions with low socio-economic conditions, like the area where this research took place (93). In this context, the most common comorbidities in participants of this study include diseases linked to chronic stress and its physiological effects, such as anxiety, sleep disorders, hypertension, and immune-mediated diseases, like allergies and autoimmune conditions. IBS, another common disorder from the group of functional GI disorders, aside from FD, is also among these frequent comorbidities. If viewed in this context, these results suggest that the characteristics of individuals with FD should be considered alongside other disorders of the gut-brain axis (94).

Another notable relationship identified in these results concerns the variable ‘Age.’ Age was initially significant in the hierarchical regression model. However, after accounting for symptoms and comorbidities, its effect was no longer evident. This finding suggests that the association between age and SWB is primarily due to health-related factors and somatization that accompanies them rather than age itself. The strong positive correlation between age and hypertension diagnosis further supports this interpretation.

In this context, a smaller subgroup of participants over 60 years of age is evident. The primary factor contributing to decreased SWB in this cohort appears to be a higher level of comorbidity and polypharmacy, rather than exposure to real-life stressors, which aligns with knowledge (94). This interpretation is supported by the stability of the variable ‘Continuous aspirin use or long-term use of NSAIDs’ in the hierarchical regression model, even after accounting for somatization and stress resilience. Although, this variable can also represent a confounding factor, due to the fact that polypharmacy, and NSAIDs in particular, may cause pathology capable of explaining the symptoms, but invisible by the standard white-light endoscopy, such as the small bowel enteropathy (95).

Ultimately, psychological factors may stay in the background of associations between increasing comorbidity, dyspepsia symptom severity, and a decline in SWB. In this term, it has been noted that older individuals with low stress resilience and insufficient adaptation to chronic diseases, that is, with psychological issues, are more likely to experience impaired SWB (96).

Among older participants, challenges in assessing somatization may arise (97). In this population, somatic symptoms related to physical illnesses, including chronic pain and fatigue, frequently overlap with symptoms of anxiety and depression, which makes difficult to differentiate these symptoms between physical and mental illnesses (98).

## 5 Conclusions, implications for practice, and future research directions

An advanced modeling procedure was applied to the group of individuals with FD referred to the gastroenterologist’s examination. The aim was to elucidate how sex interacts with associations among various FD-related factors and SWB. It has revealed a complex interactive network between age, sex, and SWB-related factors, supporting the biopsychosocial model of FD.

Psychological constructs, somatization, and stress-resilience showed the strongest associations with SWB, surpassing the effects of other variables. This result becomes reasonable, when conveyed into the concept of the gut-brain interaction, as the framework for understanding the pathophysiology of FD (Figure 1). Only the variable “chronic aspirin use or long-term NSAID use” remained significant in the final model, indicating an independent impact on SWB.

An effect of sex on SWB emerged after controlling for stress resilience, suggesting that this effect was masked by the (stronger) effect of psychological resilience on SWB, but that other sex-related factors may also (but in a lesser extent) influence SWB. For instance, women might gain more than men from certain protective psychological traits, or they employ different strategies to maintain SWB despite illness.

One such strategy, suggested by the results of this study, is that women tend to be more optimistic than men as they value positive experiences more than men do.

In contrast to women, men are likely to have a more favorable initial position regarding SWB, due to their higher stress resilience. This initial position can ultimately be hampered by the negative effect on SWB of other factors, like the use of negative health-related lifestyles, higher sensitivity to disharmonious relationships within the family, as well as a higher sensitivity to recurrent dyspeptic symptoms, and symptom broadening, as in the context of overlapping FD and IBS diagnosis. This finally supports the idea that men’s SWB may be more reactive to physical symptoms or their mental effects than women’s. In women with FD, SWB appears to be less sensitive to symptom frequency or symptom sharing with IBS, possibly due to different coping mechanisms or better tolerance of physical discomfort. These results contrast with the evidence available so far, indicating lower QL in women with FD.

Overall, the results point toward the importance of examining the gender-related (socially conditioned) factors associated with SWB or QL in the context of FD and other disorders of the gut-brain interaction. The results also indicate that there is a need to separately assess the FD-related factors and their influence on SWB in older individuals with FD, as they can differ from those in their younger counterparts.

Taken together, these results suggest that sex-specific differences in factors such as coping styles, health-seeking behaviors, emotional expression, health-related attitudes, symptom reporting, and social gender role expectations should be explored in future research and considered in the development of intervention strategies.

An individualized and multicomponent approach that considers sex issues might improve FD treatment and improve patient SWB. Interventions for men should address both symptom management and the psychological impact of symptoms, using integrated approaches (e.g., psychoeducation, coping skills training). Promoting health and emotional literacy in men may also be useful in improving their SWB.

Clinicians should expand definitions of resilience to include both internal strength (e.g., perseverance, coping styles) and interpersonal strategies (e.g., seeking support), recognizing that each may play a different role depending on sex.

However, identifying a suitable methodological framework to elucidate complex relationships within the context of the biopsychosocial model of FD remains a challenge. Current attempts to construct comprehensive models are in the early stages. The findings of this study align with the limited existing research in this area, pointing toward the need to involve psychological constructs like somatization and stress resilience in these studies, due to their strong associations with SWB.

A major limitation of this study is the lack of invasive diagnostics of HP infection, but this fact could not have had a significant impact on study results, since non-invasive SAT was taken. In addition, standard questionnaires for rating symptom severity have not been used; instead, a 5-item Likert scale was used to measure subjective participant perception of symptom intensity.

A limitation may also be a selected population of individuals with FD who were referred for a specialist examination. However, this selection enabled the analysis of complex, multilayered relationships among variables within a shared context. The study not only investigated the existence of these relationships but also examined their directionality and relative strength.

## Data Availability

The original contributions presented in the study are included in the article/Supplementary material, further inquiries can be directed to the corresponding author.

## Glossary

FD: functional dyspepsia
GI: gastrointestinal
SWB: subjective well-being
PDS: postprandial distress syndrome
EPS: epigastric pain syndrome
IBS: irritable bowel syndrome
CNS: central nervous system
ENS: enteric nervous system
QL: quality of life
NSAIDs: nonsteroidal anti-inflammatory drugs
HP: *Helicobacter pylori*
BMI: body mass index
UEG: United European Gastroenterology
ESNM: European Society for Neurogastroenterology and Motility
HPA: Hypothalamic–Pituitary–Adrenal
ANS: autonomic nervous system
SAT: stool antigen test
PAGI-SYM: Patient Assessment of Gastrointestinal Disorders-Symptom Severity Index
LPDS: Leuven Postprandial Distress Scale
PHQ: Patient Health Questionnaire
ESGE: European Society of Gastrointestinal Endoscopy

## References

[1] Sperber AD Bangdiwala SI Drossman DA Ghoshal UC Simren M Tack J . Worldwide prevalence and burden of functional gastrointestinal disorders, results of Rome foundation global study. Gastroenterology. (2021) 160:99–114.e3. doi: 10.1053/j.gastro.2020.04.014, 32294476

[2] Lee K Kwon C Yeniova AÖ Koyanagi A Jacob L Smith L . Global prevalence of functional dyspepsia according to Rome criteria, 1990–2020: a systematic review and meta-analysis. Sci Rep. (2024) 14:4172. doi: 10.1038/s41598-024-54716-3, 38378941 PMC10879214

[3] Harer KN Hasler WL. Functional dyspepsia: a review of the symptoms, evaluation, and treatment options. Gastroenterol Hepatol. (2020) 16:66–74. PMC8132673 34035704

[4] den Van Houte K Carbone F Goelen N Schol J Masuy I Arts J . Effects of Rome IV definitions of functional dyspepsia subgroups in secondary care. Clin Gastroenterol Hepatol. (2021) 19:1620–6. doi: 10.1016/j.cgh.2020.06.043 32645450

[5] Ford AC Mahadeva S Carbone MF Lacy BE Talley NJ. Functional dyspepsia. Lancet. (2020) 396:1689–702. doi: 10.1016/S0140-6736(20)30469-4 33049222

[6] Nasseri-Moghaddam S Mousavian A-H Kasaeian A Kanno T Yuan Y Ford AC . What is the prevalence of clinically significant endoscopic findings in subjects with dyspepsia? Updated systematic review and Meta-analysis. Clin Gastroenterol Hepatol. (2023) 21:1739–1749.e2. doi: 10.1016/j.cgh.2022.05.041, 35738355

[7] Koppen IJN Nurko S Saps M Di Lorenzo C Benninga MA. The pediatric Rome IV criteria: what’s new? Expert Rev Gastroenterol Hepatol. (2017) 11:193–201. doi: 10.1080/17474124.2017.1282820, 28092724

[8] Drossman DA. Functional gastrointestinal disorders: history, pathophysiology, clinical features and Rome IV. Gastroenterology. (2016) 150:S0016-5085(16)00223-7. doi: 10.1053/j.gastro.2016.02.032, 27144617

[9] Drossman DA Hasler WL. Rome IV-functional GI disorders: disorders of gut-brain interaction. Gastroenterology. (2016) 150:1257–61. doi: 10.1053/j.gastro.2016.03.035, 27147121

[10] Black CJ Drossman DA Talley NJ Ruddy J Ford AC. Functional gastrointestinal disorders: advances in understanding and management. Lancet. (2020) 396:1664–74. doi: 10.1016/S0140-6736(20)32115-2, 33049221

[11] Mayer EA Labus JS Tillisch K Cole SW Baldi P. Towards a systems view of IBS. Nat Rev Gastroenterol Hepatol. (2015) 12:592–605. doi: 10.1038/nrgastro.2015.121, 26303675 PMC5001844

[12] Appleton J. The gut-brain Axis: influence of microbiota on mood and mental health. Integr Med Encinitas Calif. (2018) 17:28–32. PMC6469458 31043907

[13] Van Oudenhove L Crowell MD Drossman DA Halpert AD Keefer L Lackner JM . Biopsychosocial aspects of functional gastrointestinal disorders. Gastroenterology. (2016):S0016-5085(16)00218-3. doi: 10.1053/j.gastro.2016.02.027 PMC8809487 27144624

[14] Stanghellini V Chan FKL Hasler WL Malagelada JR Suzuki H Tack J . Gastroduodenal Disorders. Gastroenterology. (2016) 150:1380–92. doi: 10.1053/j.gastro.2016.02.011, 27147122

[15] Ford AC Marwaha A Sood R Moayyedi P. Global prevalence of, and risk factors for, uninvestigated dyspepsia: a meta-analysis. Gut. (2015) 64:1049–57. doi: 10.1136/gutjnl-2014-307843, 25147201

[16] Li Y Gong Y Li Y He D Wu Y Wang H . Sleep disturbance and psychological distress are associated with functional dyspepsia based on Rome III criteria. BMC Psychiatry. (2018) 18:133. doi: 10.1186/s12888-018-1720-0, 29776354 PMC5960153

[17] Esterita T Dewi S Suryatenggara FG Glenardi G. Association of Functional Dyspepsia with depression and anxiety: a systematic review. J Gastrointest Liver Dis JGLD. (2021) 30:259–66. doi: 10.15403/jgld-3325, 33951117

[18] Carrozzino D Porcelli P. Alexithymia in gastroenterology and hepatology: a systematic review. Front Psychol. (2018) 9:470. doi: 10.3389/fpsyg.2018.00470, 29681874 PMC5897673

[19] Hantoro IF Syam AF Mudjaddid E Setiati S Abdullah M. Factors associated with health-related quality of life in patients with functional dyspepsia. Health Qual Life Outcomes. (2018) 16:83. doi: 10.1186/s12955-018-0913-z, 29720190 PMC5930843

[20] Singh P Ballou S Rangan V Katon J Hassan R Iturrino J . Clinical and psychological factors predict outcome in patients with functional dyspepsia: a prospective study. Clin Gastroenterol Hepatol Off Clin Pract J Am Gastroenterol Assoc. (2022) 20:1251–1258.e1. doi: 10.1016/j.cgh.2021.07.043, 34339874

[21] Duncanson KR Talley NJ Walker MM Burrows TL. Food and functional dyspepsia: a systematic review. J Hum Nutr Diet Off J Br Diet Assoc. (2018) 31:390–407. doi: 10.1111/jhn.12506 28913843

[22] Arnaout AY Alhejazi TJ Narabani Y Hamdan O Arnaout K Arnaout I . The prevalence and risk factors of functional dyspepsia among adults in low- and middle-income countries: an international cross-sectional study. Medicine (Baltimore). (2023) 102:e35437. doi: 10.1097/MD.0000000000035437 37800762 PMC10553146

[23] Wauters L Dickman R Drug V Mulak A Serra J Enck P . United European gastroenterology (UEG) and European Society of Neurogastroenterology and Motility (ESNM) consensus on functional dyspepsia. United Eur Gastroenterol J. (2021) 9:307–31. doi: 10.1002/ueg2.12061 PMC8259261 33939891

[24] Aro P Talley NJ Johansson S-E Agréus L Ronkainen J. Anxiety is linked to new-onset dyspepsia in the Swedish population: a 10-year follow-up study. Gastroenterology. (2015) 148:928–37. doi: 10.1053/j.gastro.2015.01.039, 25644097

[25] Koloski NA Jones M Talley NJ. Evidence that independent gut-to-brain and brain-to-gut pathways operate in the irritable bowel syndrome and functional dyspepsia: a 1-year population-based prospective study. Aliment Pharmacol Ther. (2016) 44:592–600. doi: 10.1111/apt.13738, 27444264

[26] Jones MP Oudenhove LV Koloski N Tack J Talley NJ. Early life factors initiate a “vicious circle” of affective and gastrointestinal symptoms: a longitudinal study. United Eur Gastroenterol J. (2013) 1:394–402. doi: 10.1177/2050640613498383, 24917988 PMC4040772

[27] Klaassen T Vork L Smeets FGM Troost FJ Kruimel JW Leue C . The interplay between stress and fullness in patients with functional dyspepsia and healthy controls: an exploratory experience sampling method study. Psychosom Med. (2022) 84:306–12. doi: 10.1097/PSY.0000000000001012 34524263

[28] Heidari Z Keshteli AH Feizi A Afshar H Adibi P. Somatic complaints are significantly associated with chronic uninvestigated dyspepsia and its symptoms: a large cross-sectional population based study. J Neurogastroenterol Motil. (2017) 23:80–91. doi: 10.5056/jnm16020, 27503912 PMC5216638

[29] Van Oudenhove L Vandenberghe J Geeraerts B Vos R Persoons P Fischler B . Determinants of symptoms in functional dyspepsia: gastric sensorimotor function, psychosocial factors or somatisation? Gut. (2008) 57:1666–73. doi: 10.1136/gut.2008.158162 18625692

[30] Agosti V Quitkin FM Stewart JW McGrath PJ. Somatization as a predictor of medication discontinuation due to adverse events. Int Clin Psychopharmacol. (2002) 17:311–4. doi: 10.1097/00004850-200211000-00007, 12409685

[31] Jerndal P Ringström G Agerforz P Karpefors M Akkermans LM Bayati A . Gastrointestinal-specific anxiety: an important factor for severity of GI symptoms and quality of life in IBS. Neurogastroenterol Motil. (2010) 22:e179:646. doi: 10.1111/j.1365-2982.2010.01493.x 20367800

[32] Tanner SE Burton Murray H Brown TA Malik Z Parkman HP. Gastrointestinal-specific symptom anxiety in patients with gastroparesis: relationships to symptom severity and quality of life. Neurogastroenterol Motil. (2023) 35:e14534. doi: 10.1111/nmo.14534, 36740788 PMC11289649

[33] Barry S Dinan TG. Functional dyspepsia: are psychosocial factors of relevance? World J Gastroenterol. (2006) 12:2701–7. doi: 10.3748/wjg.v12.i17.2701, 16718756 PMC4130978

[34] Welén K Faresjö A Faresjö T. Functional dyspepsia affects women more than men in daily life: a case-control study in primary care. Gend Med. (2008) 5:62–73. doi: 10.1016/s1550-8579(08)80009-5, 18420167

[35] Houghton LA Heitkemper M Crowell M Emmanuel A Halpert A McRoberts JA . Age, gender and women’s health and the patient. Gastroenterology. (2016) 150:S0016-5085(16)00183-9. doi: 10.1053/j.gastro.2016.02.017, 27144622

[36] Aziz I Palsson OS Törnblom H Sperber AD Whitehead WE Simrén M. Epidemiology, clinical characteristics, and associations for symptom-based Rome IV functional dyspepsia in adults in the USA, Canada, and the UK: a cross-sectional population-based study. Lancet Gastroenterol Hepatol. (2018) 3:252–62. doi: 10.1016/S2468-1253(18)30003-7, 29396034

[37] Kim YS Kim N. Functional dyspepsia: a narrative review with a focus on sex-gender differences. J Neurogastroenterol Motil. (2020) 26:322–34. doi: 10.5056/jnm20026, 32606255 PMC7329152

[38] Mulak A Taché Y Larauche M. Sex hormones in the modulation of irritable bowel syndrome. World J Gastroenterol. (2014) 20:2433–48. doi: 10.3748/wjg.v20.i10.2433, 24627581 PMC3949254

[39] Krispil-Alon M Jovasevic V Radulovic J Richter-Levin G. Sex-specific roles of hippocampal microRNAs in stress vulnerability and resilience. Transl Psychiatry. (2022) 12:503. doi: 10.1038/s41398-022-02267-4, 36473835 PMC9726879

[40] Korzan WJ Summers CH. Evolution of stress responses refine mechanisms of social rank. Neurobiol Stress. (2021) 14:100328. doi: 10.1016/j.ynstr.2021.100328, 33997153 PMC8105687

[41] Kerr P Kheloui S Rossi M Désilets M Juster R-P. Allostatic load and women’s brain health: a systematic review. Front Neuroendocrinol. (2020) 59:100858. doi: 10.1016/j.yfrne.2020.100858, 32758482

[42] Mayor E. Gender roles and traits in stress and health. Front Psychol. (2015) 6:779. doi: 10.3389/fpsyg.2015.00779, 26106354 PMC4460297

[43] Goldfarb EV Seo D Sinha R. Sex differences in neural stress responses and correlation with subjective stress and stress regulation. Neurobiol Stress. (2019) 11:100177. doi: 10.1016/j.ynstr.2019.100177, 31304198 PMC6603439

[44] Swinkels J Tilburg T van Verbakel E van Broese Groenou M Explaining the gender gap in the caregiving burden of partner caregivers J Gerontol B Psychol Sci Soc Sci 2019 74 309–317 doi: 10.1093/geronb/gbx036 28379560 PMC6327655

[45] De France K Evans GW Brody GH Doan SN. Cost of resilience: childhood poverty, mental health, and chronic physiological stress. Psychoneuroendocrinology. (2022) 144:105872. doi: 10.1016/j.psyneuen.2022.105872 35879139

[46] Gustafsson PE Janlert U Theorell T Westerlund H Hammarström A. Socioeconomic status over the life course and allostatic load in adulthood: results from the northern Swedish cohort. J Epidemiol Community Health. (2011) 65:986–92. doi: 10.1136/jech.2010.108332, 20974835

[47] Diderichsen F Hallqvist J Whitehead M. Differential vulnerability and susceptibility: how to make use of recent development in our understanding of mediation and interaction to tackle health inequalities. Int J Epidemiol. (2019) 48:268–74. doi: 10.1093/ije/dyy167, 30085114

[48] Juster R-P Seeman T Mcewen B Picard M Mahar I Mechawar N . Social inequalities and the road to allostatic load: from vulnerability to resilience.In: Cicchetti D (Ed.), Developmental Psychopathology. John Wiley & Sons, Inc. (2016):1–54. doi: 10.1002/9781119125556.devpsy408

[49] Bangasser DA Wiersielis KR. Sex differences in stress responses: a critical role for corticotropin-releasing factor. Hormones. (2018) 17:5–13. doi: 10.1007/s42000-018-0002-z, 29858858

[50] Suvarna B Suvarna A Phillips R Juster R-P McDermott B Sarnyai Z. Health risk behaviours and allostatic load: a systematic review. Neurosci Biobehav Rev. (2020) 108:694–711. doi: 10.1016/j.neubiorev.2019.12.020, 31846655

[51] Honkalampi K Kraav S-L Kerr P Juster R-P Virtanen M Hintsa T . Associations of allostatic load with sociodemographic factors, depressive symptoms, lifestyle, and health characteristics in a large general population-based sample. J Affect Disord. (2024) 350:784–91. doi: 10.1016/j.jad.2024.01.189, 38266933

[52] Hicks B Veronesi G Ferrario MM Forrest H Whitehead M Diderichsen F . Roles of allostatic load, lifestyle and clinical risk factors in mediating the association between education and coronary heart disease risk in Europe. J Epidemiol Community Health. (2021) 75:1147–54. doi: 10.1136/jech-2020-215394, 34049926 PMC8588289

[53] von Elm E Altman DG Egger M Pocock SJ Gøtzsche PC Vandenbroucke JP . The strengthening the reporting of observational studies in epidemiology (STROBE) statement: guidelines for reporting observational studies. J Clin Epidemiol. (2008) 61:344–9. doi: 10.1016/j.jclinepi.2007.11.008 18313558

[54] Katicić M Duvnjak M Kanizaj TF Krznarić Z Marusić M Mihaljević S . Croatian guidelines for diagnostics and treatments of *Helicobacter pylori* infection. Lijec Vjesn. (2014) 136:1–17. 24720149

[55] Malfertheiner P Megraud F O’Morain CA Atherton J Axon ATR Bazzoli F . Management of *Helicobacter pylori* infection--the Maastricht IV/ Florence consensus report. Gut. (2012) 61:646–64. doi: 10.1136/gutjnl-2012-302084 22491499

[56] Malfertheiner P Megraud F Rokkas T Gisbert JP Liou J-M Schulz C . Management of *Helicobacter pylori* infection: the Maastricht VI/Florence consensus report. Gut. (2022):gutjnl-2022-327745. doi: 10.1136/gutjnl-2022-327745 35944925

[57] Best LM Takwoingi Y Siddique S Selladurai A Gandhi A Low B . Non-invasive diagnostic tests for *Helicobacter pylori* infection. Cochrane Database Syst Rev. (2018) 2018:CD012080. doi: 10.1002/14651858.CD012080.pub2, 29543326 PMC6513531

[58] Brkić N Terzić V Švagelj M Cvrković M Brkić H Švagelj D. The prevalence and characteristics of *Helicobacter pylori*-associated gastritis in dyspeptic patients in eastern Croatia, determined by immunohistochemistry. Period Biol. (2017) 119:75–80. doi: 10.18054/pb.v119i1.4219

[59] Rentz AM Kahrilas P Stanghellini V Tack J Talley NJ de la Loge C . Development and psychometric evaluation of the patient assessment of upper gastrointestinal symptom severity index (PAGI-SYM) in patients with upper gastrointestinal disorders. Qual Life Res Int J Qual Life Asp Treat Care Rehabil. (2004) 13:1737–49. doi: 10.1007/s11136-004-9567-x, 15651544

[60] Carbone F Vandenberghe A Holvoet L Vanuytsel T Van Oudenhove L Jones M . Validation of the Leuven postprandial distress scale, a questionnaire for symptom assessment in the functional dyspepsia/postprandial distress syndrome. Aliment Pharmacol Ther. (2016) 44:989–1001. doi: 10.1111/apt.13753 27518319

[61] Diener E Wirtz D Biswas-Diener R Tov W Kim-Prieto C Choi D-W . New measures of well-being. In: Diener E (Ed.) assessing well-being. Soc. Indic. Res. Dordrecht: Springer, (2009) 39:247–66. doi: 10.1007/978-90-481-2354-4_12

[62] Komšo T Burić I. Dienerove skale subjektivne dobrobiti: Skala zadovoljstva životom, Skala prosperiteta i Skala pozitivnih i negativnih iskustava In: Ivanišević M. N. Macuka I. Nekić M. Ombla J. Šimunić A. (eds). Zbirka psihologijskih skala i upitnika. Zadar, Croatia: Sveučilište u Zadru (2016)

[63] MSc LR. How to use the Connor Davidson resilience scale (CD-RISC). (2019) Available online at: https://positivepsychology.com/connor-davidson-brief-resilience-scale/ [Accessed October 19, 2025]

[64] Slišković A Burić I Adorić VĆ Nikolić M Junaković IT Slišković A . *Zbirka psihologijskih skala i upitnika: svezak 9*. Morepress Books. (2020). Available online at: https://morepress.unizd.hr/books [Accessed October 19, 2025]

[65] Kocalevent R-D Hinz A Brähler E. Standardization of a screening instrument (PHQ-15) for somatization syndromes in the general population. BMC Psychiatry. (2013) 13:91. doi: 10.1186/1471-244X-13-91, 23514436 PMC3606198

[66] Ivanišević MN Macuka I Nekić M Ombla J Šimunić A Ivanišević MN . *Zbirke psihologijskih skala i upitnika: svezak 11*. Morepress Books. (2022). Available online at: https://morepress.unizd.hr/books [Accessed January 3, 2026]

[67] Liu Z Ji J Yang Q Pang F Qiu Y. Diagnostic performance of white light endoscopy, narrow band imaging, and flexible spectral imaging color enhancement for early gastric cancer: a systematic review and meta-analysis. Quant Imaging Med Surg. (2025) 15:5534–45. doi: 10.21037/qims-24-482, 40606389 PMC12209649

[68] Bisschops R Areia M Coron E Dobru D Kaskas B Kuvaev R . Performance measures for upper gastrointestinal endoscopy: a European Society of Gastrointestinal Endoscopy (ESGE) quality improvement initiative. Endoscopy. (2016) 48:843–64. doi: 10.1055/s-0042-113128, 27548885

[69] Streiner DL. Finding our way: an introduction to path analysis. Can J Psychiatr. (2005) 50:115–22. doi: 10.1177/070674370505000207, 15807228

[70] Aro P Talley NJ Agréus L Johansson S-E Bolling-Sternevald E Storskrubb T . Functional dyspepsia impairs quality of life in the adult population. Aliment Pharmacol Ther. (2011) 33:1215–24. doi: 10.1111/j.1365-2036.2011.04640.x, 21443537

[71] Chang L. Review article: epidemiology and quality of life in functional gastrointestinal disorders. Aliment Pharmacol Ther. (2004) 20:31–9. doi: 10.1111/j.1365-2036.2004.02183.x, 15521853

[72] Piazza JR Charles ST Almeida DM. Living with chronic health conditions: age differences in affective well-being. J Gerontol B Psychol Sci Soc Sci. (2007) 62:P313–21. doi: 10.1093/geronb/62.6.p313 18079415

[73] Sliwinski MJ Freed S Scott SB Pasquini G Smyth JM. Does chronic stress moderate age differences in emotional well-being? Testing predictions of strength and vulnerability integration. J Gerontol B Psychol Sci Soc Sci. (2021) 76:1104–13. doi: 10.1093/geronb/gbaa174, 33057679 PMC8200348

[74] Wittlinger T Bekić S Guljaš S Periša V Volarić M Trtica Majnarić L. Patterns of the physical, cognitive, and mental health status of older individuals in a real-life primary care setting and differences in coping styles. Front Med. (2022) 9:989814. doi: 10.3389/fmed.2022.989814, 36388902 PMC9650321

[75] Zeng F Sun R He Z Chen Y Lei D Yin T . Altered functional connectivity of the amygdala and sex differences in functional dyspepsia. Clin Transl Gastroenterol. (2019) 10:e00046. doi: 10.14309/ctg.0000000000000046, 31136362 PMC6613861

[76] Ofman JJ Maclean CH Straus WL Morton SC Berger ML Roth EA . Meta-analysis of dyspepsia and nonsteroidal antiinflammatory drugs. Arthritis Rheum. (2003) 49:508–18. doi: 10.1002/art.11192, 12910557

[77] Yap PR-Y Goh K-L. Non-steroidal anti-inflammatory drugs (NSAIDs) induced dyspepsia. Curr Pharm Des. (2015) 21:5073–81. doi: 10.2174/1381612821666150915105738, 26369685

[78] Sostres C Gargallo CJ Lanas A. Nonsteroidal anti-inflammatory drugs and upper and lower gastrointestinal mucosal damage. Arthritis Res Ther. (2013) 15 Suppl 3:S3. doi: 10.1186/ar4175, 24267289 PMC3890944

[79] Elsenbruch S. Abdominal pain in irritable bowel syndrome: a review of putative psychological, neural and neuro-immune mechanisms. Brain Behav Immun. (2011) 25:386–94. doi: 10.1016/j.bbi.2010.11.010, 21094682

[80] Jones MP Coppens E Vos R Holvoet L Luyten P Tack J . A multidimensional model of psychobiological interactions in functional dyspepsia: a structural equation modelling approach. Gut. (2013) 62:1573–80. doi: 10.1136/gutjnl-2012-302634, 22917658

[81] Van Oudenhove L Vandenberghe J Vos R Holvoet L Demyttenaere K Tack J. Risk factors for impaired health-related quality of life in functional dyspepsia. Aliment Pharmacol Ther. (2011) 33:261–74. doi: 10.1111/j.1365-2036.2010.04510.x, 21083672

[82] Yong G Wang X Xu Y He G. The influence of subjective well-being on anxiety, depression and quality of life in patients with functional dyspepsia. BMC Gastroenterol. (2025) 25:490. doi: 10.1186/s12876-025-04075-8, 40597742 PMC12211792

[83] Hoang D Kristoffersen I Li IW. All in the mind? Estimating the effect of mental health on health behaviours. Soc Sci Med. (2019) 225:69–84. doi: 10.1016/j.socscimed.2019.02.017, 30818089

[84] Cotman CW Berchtold NC Christie L-A. Exercise builds brain health: key roles of growth factor cascades and inflammation. Trends Neurosci. (2007) 30:464–72. doi: 10.1016/j.tins.2007.06.011, 17765329

[85] Ashoorion V Hosseinian S-Z Rezaei N Hajihashemi P Zare-Farashbandi E Adibi P. Effect of dietary patterns on functional dyspepsia in adults: a systematic review. J Health Popul Nutr. (2025) 44:132. doi: 10.1186/s41043-025-00884-5, 40270080 PMC12020029

[86] Volarić M Šojat D Majnarić LT Vučić D. The association between functional dyspepsia and metabolic syndrome-the state of the art. Int J Environ Res Public Health. (2024) 21:237. doi: 10.3390/ijerph21020237, 38397726 PMC10888556

[87] Koloski NA Talley NJ Boyce PM. Predictors of health care seeking for irritable bowel syndrome and nonulcer dyspepsia: a critical review of the literature on symptom and psychosocial factors. Am J Gastroenterol. (2001) 96:1340–9. doi: 10.1111/j.1572-0241.2001.03789.x, 11374666

[88] Narayanan SP Anderson B Bharucha AE. Sex- and gender-related differences in common functional Gastroenterologic disorders. Mayo Clin Proc. (2021) 96:1071–89. doi: 10.1016/j.mayocp.2020.10.004, 33814075 PMC8075061

[89] Billey A Saleem A Zeeshan B Dissanayake G Zergaw MF Elgendy M . The bidirectional relationship between sleep disturbance and functional dyspepsia: a systematic review to understand mechanisms and implications on management. Cureus. (2024) 16:e66098. doi: 10.7759/cureus.66098, 39229406 PMC11370990

[90] Filipović BF Randjelovic T Ille T Markovic O Milovanović B Kovacevic N . Anxiety, personality traits and quality of life in functional dyspepsia-suffering patients. Eur J Intern Med. (2013) 24:83–6. doi: 10.1016/j.ejim.2012.06.017, 22857883

[91] Mariotti A. The effects of chronic stress on health: new insights into the molecular mechanisms of brain-body communication. Future Sci OA. (2015) 1:FSO23. doi: 10.4155/fso.15.21, 28031896 PMC5137920

[92] Volarić N Šojat D Volarić M Včev I Keškić T Majnarić LT. The gender and age perspectives of allostatic load. Front Med. (2024) 11:1502940. doi: 10.3389/fmed.2024.1502940, 39741506 PMC11685202

[93] Crielaard L Nicolaou M Sawyer A Quax R Stronks K. Understanding the impact of exposure to adverse socioeconomic conditions on chronic stress from a complexity science perspective. BMC Med. (2021) 19:242. doi: 10.1186/s12916-021-02106-1, 34635083 PMC8507143

[94] Šojat D Volarić M Keškić T Volarić N Cerovečki V Trtica Majnarić L. Putting functional gastrointestinal disorders within the spectrum of inflammatory disorders can improve classification and diagnostics of these disorders. Biomedicine. (2024) 12:702. doi: 10.3390/biomedicines12030702, 38540315 PMC10967747

[95] Lee IS Cho YK. Initial steps to prevent nonsteroidal anti-inflammatory Drug- or aspirin-induced enteropathy: long-term outcome data. Gut Liver. (2015) 9:697–8. doi: 10.5009/gnl15287, 26503568 PMC4625695

[96] Majnarić LT Bosnić Z Guljaš S Vučić D Kurevija T Volarić M . Low psychological resilience in older individuals: an association with increased inflammation, oxidative stress and the presence of chronic medical conditions. Int J Mol Sci. (2021) 22:8970. doi: 10.3390/ijms22168970, 34445675 PMC8396457

[97] Drayer RA Mulsant BH Lenze EJ Rollman BL Dew MA Kelleher K . Somatic symptoms of depression in elderly patients with medical comorbidities. Int J Geriatr Psychiatry. (2005) 20:973–82. doi: 10.1002/gps.1389, 16163749

[98] Majnarić LT Bekić S Babič F Pusztová Ľ Paralič J. Cluster analysis of the associations among physical frailty, cognitive impairment and mental disorders. Med Sci Monit. (2020) 26:e924281. doi: 10.12659/MSM.924281, 32929055 PMC7518080

