# Supplementary material for: Factors influencing subjective well-being in individuals with functional dyspepsia — a path analysis of sex and psychological factors
Source: Front Med (Lausanne). 2026 Jan 30;13:1728748. doi: 10.3389/fmed.2026.1728748 (PMC12903126; doi:10.3389/fmed.2026.1728748)
Supplement: Supplementary file 1 [file Table_1.docx]

Supplementary Material 1

**1 Reliability analysis – internal consistency of the scale**

**Supplementary Table.** Reliability analysis – internal consistency of the scale.

| Item | *r* item-total | α (if the item is ejected) |
| --- | --- | --- |
| 1 | 0,61 | 0,75 |
| 2 | 0,56 | 0,75 |
| 3 | 0,65 | 0,73 |
| 4 | 0,68 | 0,73 |
| 5 | 0,65 | 0,73 |
| 6 | 0,61 | 0,74 |
| 7 | 0,41 | 0,76 |
| 8 | 0,68 | 0,73 |
| 9 | 0,44 | 0,77 |

Legend: *r* item-total – correlation of the item with the total scale

In the table above, all item-total correlations are above 0.3, which is considered desirable. The internal consistency of the scale, measured by Cronbach's alpha, is 0.77. A Cronbach's alpha greater than 0.7 is generally deemed acceptable for research purposes, while values above 0.8 are regarded as good, indicating strong internal consistency.

In a validation study conducted on the Croatian population by Slišković et al. (2022), the PHQ-15 questionnaire was validated using two very homogeneous and healthy samples: men (N = 284) and women (N = 466). The internal consistency for the men's group was 0.75, while the women's group had an internal consistency of 0.80.

**2 Criterion validity of the somatization scale**

We assessed the criterion validity by examining the correlation between the somatization symptom scale and Diener's SWB Measures and the Stress Resilience Scale.

The somatization scale shows a negative correlation with the life satisfaction scale (r = -0.42), the prosperity scale (r = -0.33), and the positive experiences scale (r = -0.38). It has a positive correlation with the negative experiences scale (r = 0.44). All of these correlations are significant at p < 0.001. Additionally, these findings align with those from a validation study conducted in Croatia by Slišković et al. (2022).

Moreover, the somatization scale is moderately negatively correlated with the stress resilience scale (r = -0.42). We expect stress resilience to be negatively correlated with the level of somatization symptoms, which further supports the criterion validity of the scale (see Figure 1).


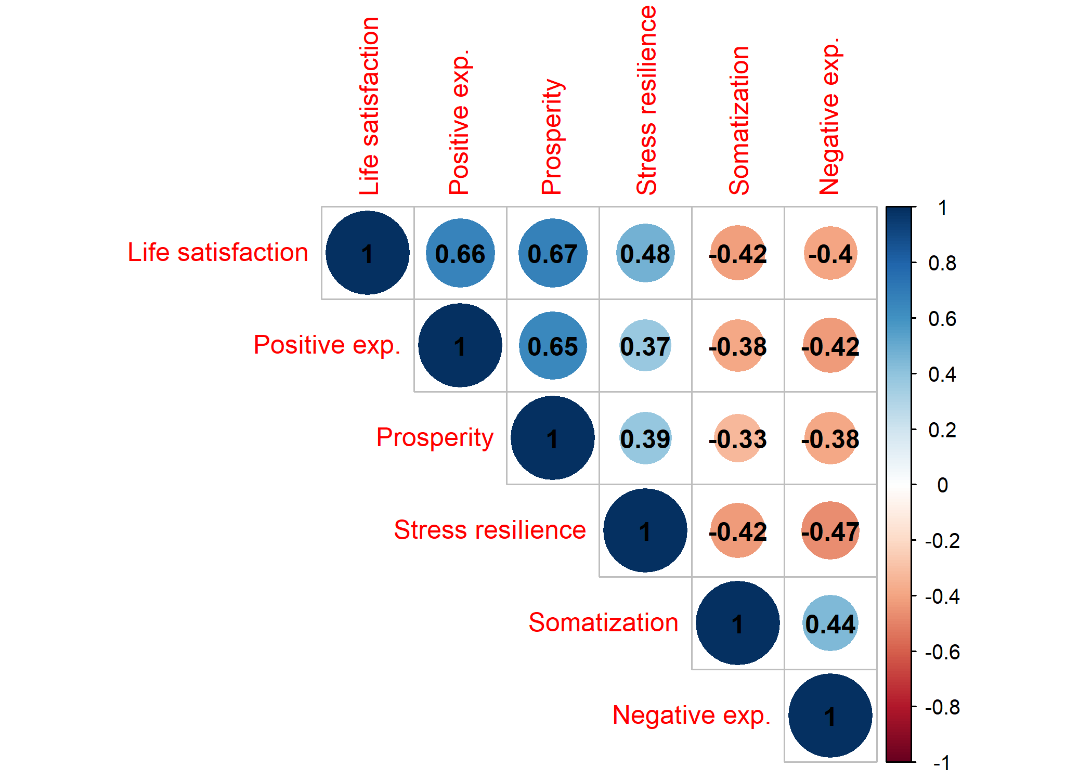


**Supplementary Figure.** Correlation matrix of the scales used (LSL: Life Satisfaction Scale)

According to Diener (1984), SWB encompasses two key components: life satisfaction, which is cognitive in nature, and the balance of positive and negative affect. Prosperity extends beyond this definition, incorporating an eudaimonic perspective on SWB. The correlations observed are consistent with expectations, and the strongest correlations are found with the Diener scales, as they measure a common concept: SWB.
