# Supplementary material for: Factors influencing subjective well-being in individuals with functional dyspepsia — a path analysis of sex and psychological factors
Source: Front Med (Lausanne). 2026 Jan 30;13:1728748. doi: 10.3389/fmed.2026.1728748 (PMC12903126; doi:10.3389/fmed.2026.1728748)
Supplement: Supplementary file 2 [file Table_2.docx]

Supplementary Material 2

**1 The relative frequencies of responses on the Somatization Symptom Questionnaire by**

**the participant sex**

**Supplementary Table.** Relative frequencies of responses on the Somatization Questionnaire.

|  | Men (n = 76) | | | Women (n = 114) | | |
| --- | --- | --- | --- | --- | --- | --- |
|  | Not bothered at all | Bothered a little | Bothered a lot | Not bothered at all | Bothered a little | Bothered a lot |
| Chronic or frequent pain in the abdomen and pelvis, back, arms, legs, or joints | 35.5% | 32.9% | 31.6% | 35.7% | 43.5% | 20.9% |
| Headaches | 46.1% | 48.7% | 5.3% | 33.0% | 51.3% | 15.7% |
| Chest pain | 75.0% | 23.7% | 1.3% | 68.7% | 25.2% | 6.1% |
| Sensation of throat tightness with breathing difficulties | 53.9% | 43.4% | 2.6% | 61.7% | 26.1% | 12.2% |
| Sensation of palpitations or rapid heartbeat | 50.0% | 48.7% | 1.3% | 48.7% | 38.3% | 13.0% |
| Dizziness | 64.5% | 34.2% | 1.3% | 53.0% | 36.5% | 10.4% |
| Fainting or loss of consciousness | 90.8% | 9.2% | 0.0% | 84.3% | 13.9% | 1.7% |
| Feeling of fatigue or lack of energy | 22.4% | 48.7% | 28.9% | 16.5% | 53.0% | 30.4% |
| Sleep problems | 51.3% | 43.4% | 5.3% | 48.2% | 41.2% | 10.5% |
