# Supplementary material for: Factors influencing subjective well-being in individuals with functional dyspepsia — a path analysis of sex and psychological factors
Source: Front Med (Lausanne). 2026 Jan 30;13:1728748. doi: 10.3389/fmed.2026.1728748 (PMC12903126; doi:10.3389/fmed.2026.1728748)
Supplement: Supplementary file 3 [file Table_3.docx]

Supplementary Material 3

**1 Stepwise regression analyses by conceptual blocks**

**1.1 Socio-demographic variables chosen to enter the regression**

- Age
- Sex
- Family relations
- Number of stressful events in the last 6-12 months
- Waist circumference

**Supplementary Table.** Final predictors retained in the stepwise regression model for the socio-demographic conceptual block

|  | **Subjective wellbeing** | | | |
| --- | --- | --- | --- | --- |
| *Predictors* | Estimate | SE | t value | *p* |
| (Intercept) | 1.45 | 0.76 | 1.89 | 0.060 |
| Age | -0.05 | 0.01 | -4.04 | **<0.001** |
| Conflicted family relationships (ref. Yes) – No | 1.29 | 0.52 | 2.48 | **0.014** |
| Number of stressful events | -0.64 | 0.27 | -2.37 | **0.019** |
| Sample | 177 | | | |
| R^2^ / R^2^ adj. | 0.165 / 0.151 | | | |

Within the socio-demographic block, higher age and a greater number of stressful life events were associated with lower SWB (both p < 0.05). In contrast, participants reporting no conflicted family relationships showed significantly higher SWB compared with those who did (p = 0.014). The model explained approximately 15% of the variance in SWB (R² = 0.165, adjusted R² = 0.151).

**1.2 Lifestyle and health-related habits – variables chosen to enter the regression**

- **Smoking**
- Alcohol consumption
- Working night shifts
- **Sedentary lifestyle**
- Number of unhealthy dietary habits
- Number of meal timing irregularities
- **Continuous aspirin use or long-term NSAID use**
- number of medications

**Supplementary Table**. Final predictors retained in the stepwise regression model for the lifestyle and health-related habits conceptual block

|  | **Subjective wellbeing** | | | |
| --- | --- | --- | --- | --- |
| *Predictors* | Estimate | SE | t value | *p* |
| (Intercept) | -2.22 | 0.57 | -3.89 | **<0.001** |
| Continuous aspirin use or long-term NSAID use – No | 1.58 | 0.42 | 3.75 | **<0.001** |
| Sedentary lifestyle (ref. Yes) – No | 1.11 | 0.38 | 2.91 | **0.004** |
| Smoker No | 0.84 | 0.34 | 2.49 | **0.014** |
| Number of medications | -0.26 | 0.13 | -1.89 | 0.060 |
| Sample | 177 | | | |
| R^2^ / R^2^ adj. | 0.236 / 0.218 | | | |

Within the lifestyle and health-related block, participants who did not use aspirin or NSAIDs continuously, those without a sedentary lifestyle, and non-smokers reported significantly higher SWB (all p < 0.05). The number of medications showed a negative but nonsignificant association with SWB (p = 0.060). The model accounted for approximately 22% of the variance in SWB (R² = 0.236, adjusted R² = 0.218).

**1.3 GI symptoms – variables chosen to enter the regression:**

- Symptom intensity
- Symptom frequency
- Symptom duration
- Number of alarm symptoms
- Number of other GI symptoms

**Supplementary Table**. Final predictors retained in the stepwise regression model for the GI symptoms conceptual block

|  | **Subjective wellbeing** | | | |
| --- | --- | --- | --- | --- |
| *Predictors* | Estimate | SE | t value | *p* |
| (Intercept) | 3.06 | 0.67 | 4.55 | **<0.001** |
| Symptom frequency | -0.53 | 0.15 | -3.45 | **0.001** |
| Symptom duration | -0.35 | 0.15 | -2.37 | **0.019** |
| Number of other GI symptoms | -0.17 | 0.11 | -1.48 | 0.142 |
| Sample | 177 | | | |
| R^2^ / R^2^ adj. | 0.119 / 0.104 | | | |

Within the GI symptom block, higher symptom frequency and longer symptom duration were significantly associated with lower SWB (both p < 0.05). This model explained about 10% of the variance in SWB (R² = 0.119, adjusted R² = 0.104).

**1.4 GI and extra-GI comorbidities – variables chosen to enter the regression:**

- Number of GI diseases
- IBS
- Number of somatic and neurological comorbidities
- Hypertension
- Anxiety

**Supplementary Table**. Final predictors retained in the stepwise regression model for the comorbidities conceptual block

|  | **Subjective wellbeing** | | | |
| --- | --- | --- | --- | --- |
| *Predictors* | Estimate | SE | t value | *p* |
| (Intercept) | -3.30 | 0.66 | -4.98 | **<0.001** |
| Anxiety (ref. Yes) – No | 1.74 | 0.38 | 4.63 | **<0.001** |
| Hypertension (ref. Yes) - No | 1.18 | 0.39 | 3.06 | **0.003** |
| IBS (ref. Yes) - Ne | 1.21 | 0.57 | 2.12 | **0.035** |
| Number of other GI diseases | 0.53 | 0.33 | 1.60 | 0.111 |
| Sample | 177 | | | |
| R^2^ / R^2^ adj. | 0.199 / 0.180 | | | |

Within the comorbidity block, the absence of anxiety, hypertension, and irritable bowel syndrome (IBS) was significantly associated with higher SWB (all p < 0.05). The model explained approximately 18% of the variance in SWB (R² = 0.199, adjusted R² = 0.180).

**1.5 Psychological factors chosen to enter the regression:**

- Somatization
- Stress resilience

**Supplementary Table**. Final predictors retained in the stepwise regression model for the psychological conceptual block

|  | **Subjective wellbeing** | | | |
| --- | --- | --- | --- | --- |
| *Predictors* | Estimate | SE | t value | *p* |
| (Intercept) | -3.46 | 0.92 | -3.75 | **<0.001** |
| Stress resilience | 1.58 | 0.25 | 6.24 | **<0.001** |
| Somatization | -0.25 | 0.05 | -5.26 | **<0.001** |
| Sample | 177 | | | |
| R^2^ / R^2^ adj. | 0.392 / 0.385 | | | |

Within the psychological factors block, higher stress resilience was strongly associated with greater SWB (p < 0.001), while higher somatization scores were significantly associated with lower SWB (p < 0.001). This model accounted for approximately 39% of the variance in SWB (R² = 0.392, adjusted R² = 0.385).
