# Supplementary material for: Factors influencing subjective well-being in individuals with functional dyspepsia — a path analysis of sex and psychological factors
Source: Front Med (Lausanne). 2026 Jan 30;13:1728748. doi: 10.3389/fmed.2026.1728748 (PMC12903126; doi:10.3389/fmed.2026.1728748)
Supplement: Supplementary file 4 [file Table_4.docx]

Supplementary Material 4

**1 Path analysis**

The path model shows that sex has a significant direct effect on SWB when controlling for psychological resilience to stress. However, the indirect effect through resilience is negative and significant, suppressing the overall effect. This is a classic example of a suppression effect: psychological resilience masks part of the effect of sex on SWB, which only becomes apparent when resilience is included in the model.


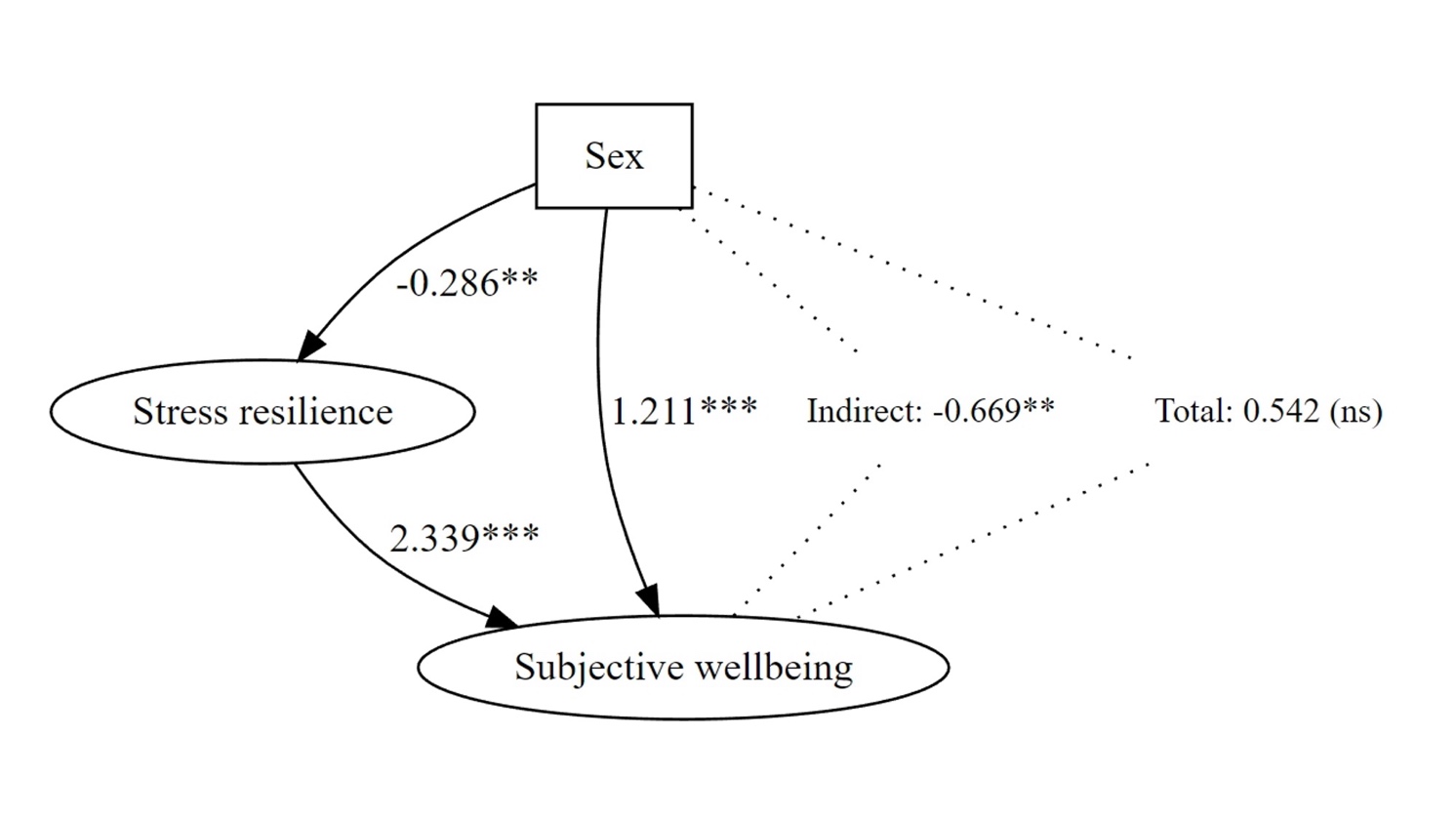


**Supplementary Figure.**

Legend: Direct effect = 1.211*, indirect effect = -0.669**, total effect = 0.542 (insignificant). A significant indirect effect suppresses the direct effect, making the total effect insignificant - a classic example of a suppression effect.
