# Supplementary material for: Factors influencing subjective well-being in individuals with functional dyspepsia — a path analysis of sex and psychological factors
Source: Front Med (Lausanne). 2026 Jan 30;13:1728748. doi: 10.3389/fmed.2026.1728748 (PMC12903126; doi:10.3389/fmed.2026.1728748)
Supplement: Supplementary file 5 [file Table_5.docx]

Supplementary Material 5

**1 The moderating effect of sex on the association between symptom frequency and subjective well-being**

A two-way ANOVA examined the effects of sex and FD symptom frequency on SWB. There was a significant main effect of symptom frequency, F (1,177) = 12.78, p = .0005, η_g_² = .067, indicating that higher symptom frequency was associated with lower subjective well-being. The main effect of sex was not significant, F (1,177) = 1.11, p = .293, η² = .006. However, a significant interaction between sex and symptom frequency emerged, F (1,177) = 4.09, p = .045, η² = .023, representing a small-to-moderate effect. As shown in the figure below, SWB declined more sharply with increasing symptom frequency among men compared to women, suggesting that more frequent symptoms may have a stronger negative impact on SWB in men.


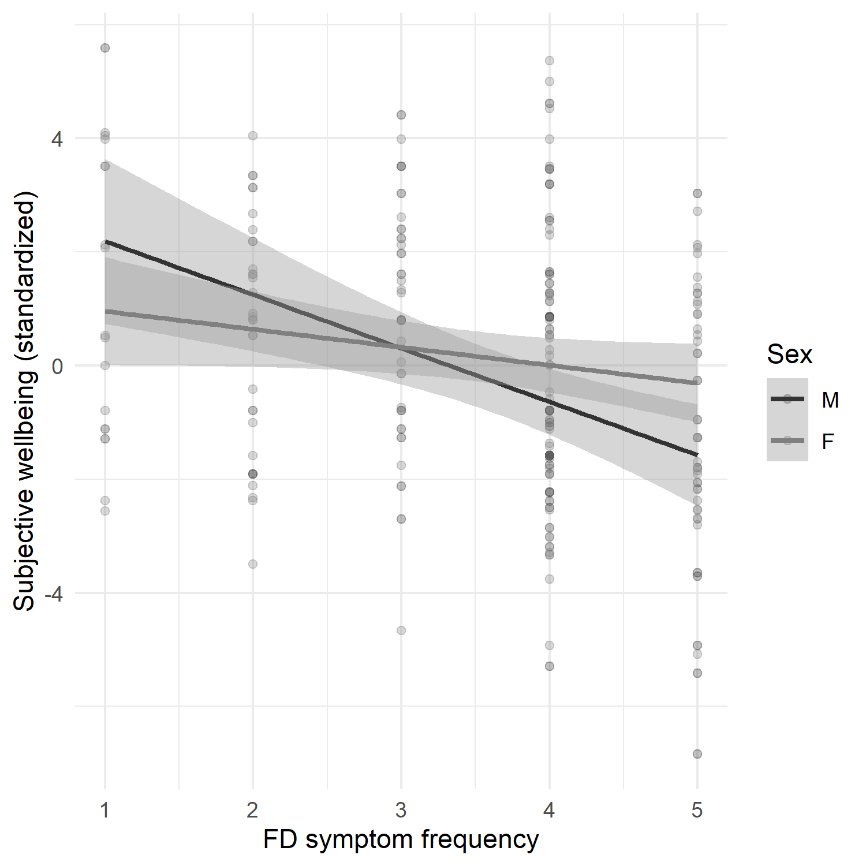


**Supplementary Figure.**

**2 Examining the effect of IBS diagnosis and sex on Subjective Life Satisfaction (the main component of SWB construct) using two-way ANOVA**

A two-way ANOVA examined the effects of sex and IBS diagnosis on Life Satisfaction Scale scores. The main effect of sex was not significant, F (1,182) = 0.16, p = .688, η² = .001, indicating no overall difference in life satisfaction between men and women. The main effect of IBS diagnosis was significant, F (1,182) = 6.69, p = .010, η² = .035, suggesting that participants with IBS reported lower life satisfaction compared to those without IBS. Importantly, a significant interaction between sex and IBS diagnosis emerged, F (1,182) = 5.47, p = .020, η² = .029, reflecting a small effect size. As shown in the figure below, the reduction in life satisfaction associated with IBS was more pronounced among men than among women.


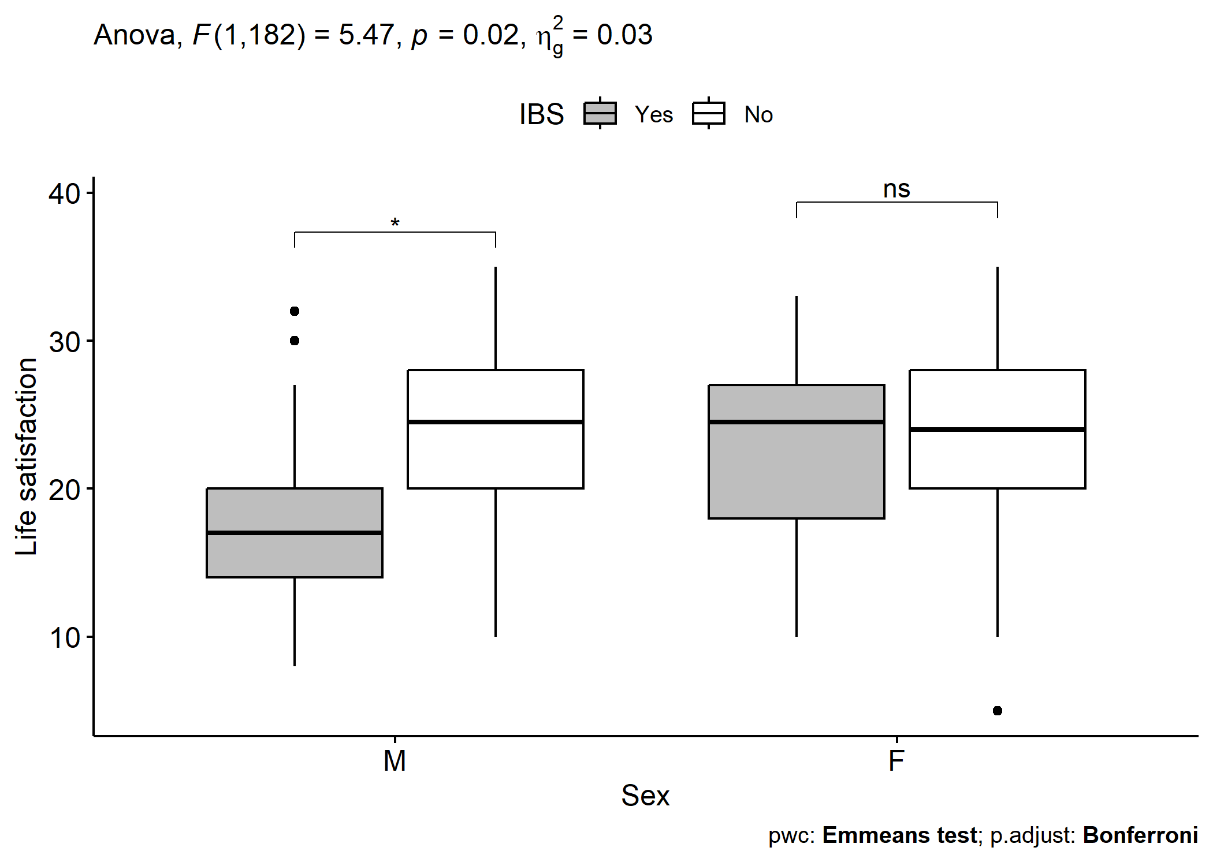


**Supplementary Figure.**
